# Ethanolphilic lactic acid bacterium *Fructilactobacillus fructivorans* as the key microorganism for fermentation of narazuke, a traditional Japanese preserved food

**DOI:** 10.1128/aem.01730-25

**Published:** 2025-11-18

**Authors:** Motomu Yoshioka, Naoki Akasaka, Yukihiko Masuda, Mariko Mori, Daisuke Watanabe

**Affiliations:** 1 Laboratory of Microbial Interaction, Division of Biological Science, Graduate School of Science and Technology, Nara Institute of Science and Technology 98337 , Ikoma, Nara, Japan; 2 Naraya Honten, Nara, Japan; 3 Morinaraduketen Co., Ltd., Nara, Japan; Universita degli Studi di Napoli Federico II, Portici, Italy

**Keywords:** narazuke, *Fructilactobacillus fructivorans*, lactic acid bacteria, ethanolphilicity, fermented food

## Abstract

Narazuke is a traditional Japanese preserved food produced by repeatedly embedding salted vegetables in sake kasu, which is a byproduct remaining after pressing sake mash. While the prototype of narazuke existed as early as the 8th century and has long been enjoyed by Japanese people, whether microbial fermentation occurs during narazuke-making remained unclear. We first analyzed the microbiota of narazuke throughout manufacturing processes, showing that the prokaryotic microbial community gradually converged and was finally dominated by a single lactic acid bacterium (LAB), *Fructilactobacillus fructivorans*. Laboratory-scale narazuke-making tests also demonstrated that *F. fructivorans* dominated the niche, with a concomitant increase in lactic acid after incubation for 2 months, revealing that the LAB was responsible for narazuke fermentation. The environment of narazuke-making is quite harsh for microbial survival because of its high ethanol content, suggesting that *F. fructivorans* proliferating in narazuke has potent ethanol tolerance. Thus, we isolated LAB strains from narazuke and investigated the effect of ethanol on their growth. The isolated narazuke strains grew even in the presence of 15% ethanol, whereas the type strain didn’t grow. The narazuke strains exhibited significantly faster growth in media containing 5%–10% ethanol than in ethanol-free medium, which is known as ethanolphilicity. Taken together, this study identified *F. fructivorans* as the main fermenting microbe in narazuke. Its abundance in an ethanol-rich environment is attributed to ethanolphilicity, which is characterized by active growth in the presence of ethanol, highlighting a novel microbial strategy for adaptation to a niche with high ethanol content.

**IMPORTANCE:**

Ethanolphilicity is a notable microbial trait that is distinct from ethanol tolerance because ethanolphilic microorganisms exhibit more active growth in the presence of ethanol than in an ethanol-free milieu. Although the mechanisms of ethanol tolerance have been intensively investigated in various microorganisms, the molecular basis underlying the effects of ethanolphilicity remains elusive. In the current study, we isolated the ethanolphilic LAB *Fructilactobacillus fructivorans* from traditional Japanese preserved food. Comparative transcriptomic analysis suggested that altered fatty acid metabolism may be involved in ethanolphilicity. Ethanolphilicity is induced by ethanol as well as other alcohols, including methanol and isopropanol. Because industrial fermentation processes are usually performed in the presence of various alcohols and solvents, which often prohibit microbial growth, our findings are expected to be applicable for developing robust strains with ethanolphilicity, as well as novel enzymes working in solvents.

## INTRODUCTION

Fermented vegetables produced using microorganisms provide various benefits to consumers, such as extended shelf life, improved food quality, and pleiotropic health-promoting effects (1). Industrially, manufacturers inoculate specific pre-cultured microbial strains into ingredients for the efficient and stable mass production of fermented foods, including fermented vegetables (2, 3). In contrast, traditional foods are spontaneously fermented by diverse microorganisms derived from ingredients and the atmosphere (1), which constitute complex microbial communities and generate a variety of metabolites that contribute to aroma, flavor, and functionalities during fermentation processes (4). Among these microbes, lactic acid bacteria (LAB), such as those belonging to the genera *Leuconostoc*, *Lactiplantibacillus*, *Pediococcus*, and *Weissella*, are the major microorganisms found in fermented vegetables (1). LAB acidify the fermentation environment by accumulating lactic acid, which inhibits the growth of other contaminating microorganisms and improves the safety of fermented foods (5). Furthermore, LAB contribute to the development of favorable flavors during fermentation (6). Sauerkraut, a representative fermented vegetable mainly favored in Germany, is a pickled cabbage fermented by various LAB, including *Leuconostoc mesenteroides*, *Lactiplantibacillus plantarum*, and *Pediococcus pentosaceus* (7). Metagenomic analysis demonstrated the succession of microbiota during fermentation; LAB in the genus *Leuconostoc* are dominant at the early stage of fermentation, and the microbial community is gradually occupied by other LAB in the genus *Lactiplantibacillus*, which previously belonged to the genus *Lactobacillus*, in parallel with fermentation progression (8). A similar tendency in the transition of microbial populations has been observed in kimchi, a traditional Korean fermented vegetable (9). Recent studies have demonstrated that several LAB isolated from kimchi produce γ-aminobutyric acid, which exhibits an anti-anxiety effect (10). Certain LAB strains from sauerkraut have gained great attention because of their applicability as probiotics (11). However, some LAB are known to be involved in food spoilage (12). For instance, the ethanol-tolerant LAB *Lactobacillus acetotolerans* and *Fructilactobacillus fructivorans* compromise the quality of sake, a traditional Japanese rice wine, by increasing acidity through lactic acid production and yielding unpleasant odors (diacetyl, acetoin, etc.) (13). These findings highlight that LAB play crucial roles in determining the quality of fermented foods, including fermented vegetables.

**Fig 1:**
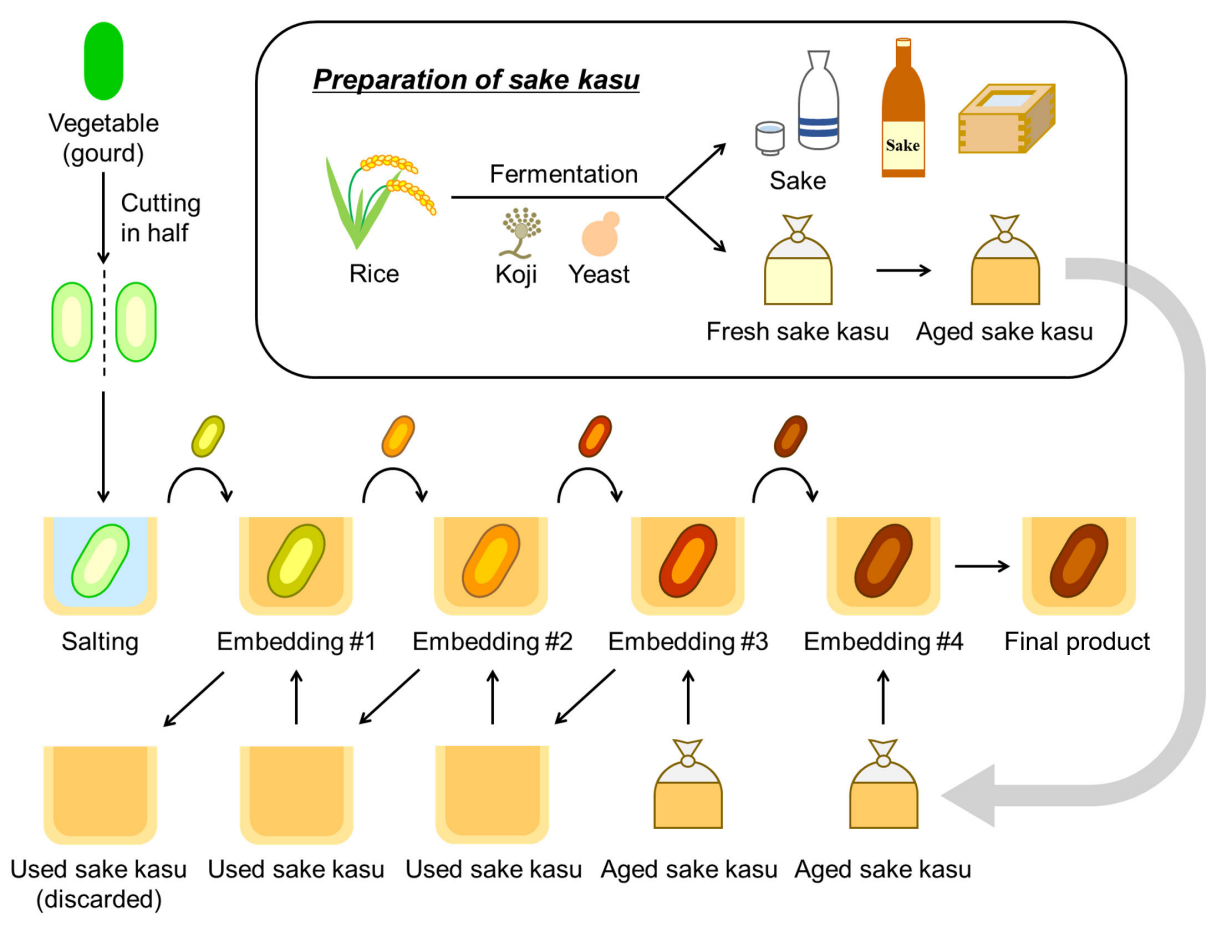
Overview of the orthodox narazuke-making method. Sake is produced by fermenting steamed rice using the koji fungus *A. oryzae* and the yeast *S. cerevisiae*, and sake kasu is a byproduct of sake fermentation remaining after pressing sake mash. Fresh sake kasu is then aged for narazuke-making. Salted vegetables are repeatedly transferred into aged sake kasu, during which salt content is drawn out of the vegetables, and the flavors of sake kasu are absorbed into them. Sake kasu used in embedding processes #2 and #3 is reused in the embedding processes #1 and #2, respectively, for the next production.

Narazuke is a traditional Japanese preserved food produced from salted gourds or other vegetables and aged sake kasu as ingredients (14). Sake kasu is a byproduct that remains after pressing the mash of sake and is widely used as a marinade base to enhance flavor and preserve food (15). In orthodox narazuke-making, vegetables are first exposed to a saturated salt solution and then repeatedly embedded in aged sake kasu, which is richer in pleasant flavors than new sake kasu and contains >8% ethanol, for a year or more (Fig. 1) (15). The ingredients are gradually desalted and instead absorb ethanol and flavors from sake kasu during the manufacturing process. The resultant narazuke (final product) generally contains 5%–8% NaCl and 6%–10% ethanol, both of which largely contribute to the development of the profound and unique flavors of narazuke, together with other aroma/flavor compounds. Narazuke has long been enjoyed by Japanese people for over 1,000 years since its prototype was established by the early 8th century (https://www3.pref.nara.jp/foodculture/index.htm). Although narazuke is one of the most popular traditional foods in Japan, it remains unclear whether microbial fermentation occurs in a manner similar to that in other fermented vegetables. A few previous studies have reported that several halotolerant yeasts and LAB, such as those in the genera *Saccharomyces* and *Lactiplantibacillus*, respectively, were isolated from narazuke (16, 17). However, it is yet to be determined whether these microorganisms actually grow and participate in the fermentation process during narazuke-making in extremely harsh environments with high salt and ethanol contents. In the present study, we first analyzed the microbiomes of narazuke samples (ingredients [salted vegetables and aged sake kasu], in-process products, and final products) (Fig. 1) collected from manufacturers to clarify the microbial dynamics during narazuke-making. Next, laboratory-scale narazuke fermentation tests were conducted by embedding the in-process products in aged sake kasu, and the microbial populations in the resultant final products were investigated. 16S ribosomal RNA (rRNA) amplicon sequencing revealed that the ethanol-tolerant LAB *F. fructivorans* dominated the microbial community in samples from both manufacturer and laboratory-scale tests. The *F. fructivorans* strain isolated from narazuke exhibited high ethanolphilicity, which is a distinct characteristic that requires a high concentration of ethanol for active growth.

## RESULTS

### Microbiota in narazuke

As described in the Introduction section, orthodox narazuke is produced by repeatedly embedding salted vegetables in aged sake kasu (Fig. 1). This suggests that the microbiota in narazuke is composed of microorganisms tolerant to harsh environments with high salt and ethanol contents. To clarify the overall microbial community in narazuke throughout the manufacturing process, we investigated the microbiome of the ingredients (salted vegetables and aged sake kasu), in-process products at the early (embedding process #2) and late (embedding process #3) stages, and final products (Fig. 1) using amplicon sequencing analyses targeting the internal transcribed spacer region 1 (ITS1) for fungi and the V4 region of the 16S rRNA gene for bacteria and archaea. Samples were collected from two manufacturers (factories N and M) that continue to produce narazuke using the orthodox method. Narazuke samples produced using uncommon methods in factories A, B, and C were also analyzed.

**Fig 2:**
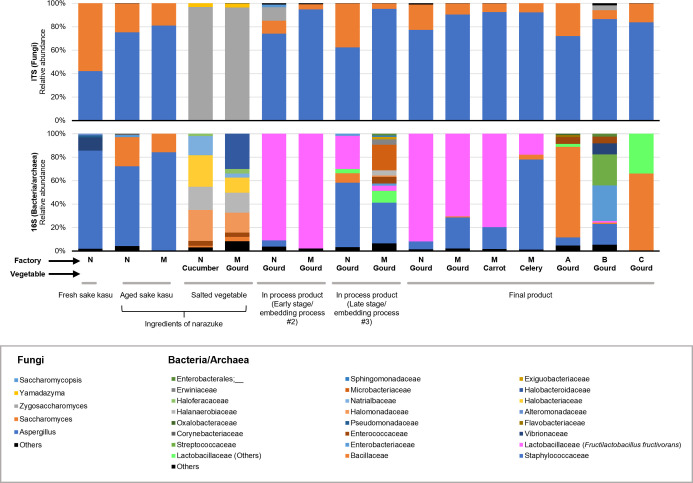
Microbiota during narazuke-making. The bars show relative abundance within each sample. The upper and lower graphs represent fungal ITS1 data at the genus level and bacterial/archaeal 16S rRNA gene data at the family level, respectively. The narazuke samples, ingredients (salted vegetables and sake kasu), in-process products, and final products were provided by the factories N and M. The commercially available final products produced by the factories A, B, and C were also subjected to amplicon sequencing.

Results showed that the metagenomic profiles of the samples from factories N and M were quite similar, suggesting a common principle in the reproducible construction of microbial ecosystems in sake kasu environments. ITS1 sequencing revealed that fungi in the genera *Aspergillus* and *Saccharomyces* occupied the microbial community of aged sake kasu, in-process products, and final products (Fig. 2). These microorganisms are thought to originate during sake fermentation (Fig. 1). In the salted vegetables, yeasts of the genera *Zygosaccharomyces* and *Yamadazyma* were detected at relative abundances of over 90% and 1%, respectively (Fig. 2). The frequency of these species sharply decreased in the subsequent manufacturing step (embedding process #2), and the corresponding DNA was barely detected in the in-process products at the later stage (embedding process #3) and in the final products (Fig. 2). As for fungal microbiota in the final products from factories A, B, and C, fungi belonging to the genera *Aspergillus* and *Saccharomyces* were the major microorganisms, similar to the samples from factories N and M (Fig. 2). To investigate whether the detected microorganisms were present in a viable state, we attempted to isolate the fungal species. Although *Saccharomyces cerevisiae* strains were isolated from fresh sake kasu, no fungi of the genera *Aspergillus* and *Saccharomyces* were obtained from aged sake kasu, in-process products, or final products (Table S1). These results suggested that the fungi detected in the ITS1 amplicon sequencing died during sake kasu aging. As for salted vegetables, the halotolerant yeasts *Zygosaccharomyces rouxii*, *Wickerhamomyces subpelliculosus*, and *Wickerhamiella versatilis* were also isolated from the samples (Table S1), all of which are known to be involved in the production of fermented products with high salt content, including soy sauce and soybean paste (18–21).

16S rRNA gene sequencing showed that bacteria in the Staphylococcaceae and Bacillaceae families were dominant in aged sake kasu and that various halotolerant bacteria and archaea (e.g., Halomonadaceae and Halobacteriaceae) were detected in salted vegetables (Fig. 2). In contrast, over 90% and 70% of the microbiota in the in-process products (embedding process #2) and final products, respectively, were composed of a single LAB, *F. fructivorans* belonging to the family Lactobacillaceae (Fig. 2). The abundance of *F. fructivorans* temporarily decreased in the in-process products at the late stage (embedding process #3) owing to the addition of new aged sake kasu, and its abundance recovered in the final products collected from factories N and M (Fig. 2). These results strongly suggested that *F. fructivorans* grew in sake kasu. Consistent with this speculation, the dominance of *F. fructivorans* was lower in narazuke made from leafy celery than in those made from other vegetables likely due to the short embedding period (embedding period [days]: leafy celery, 18; gourd, 50; and carrot, 74) (Fig. 2). We then attempted to isolate microorganisms from the narazuke samples collected from factories N and M. When De Man-Rogosa-Sharpe (MRS) agar was used as a medium for isolation, only four of 22 isolates were identified as *F. fructivorans* based on the partial sequence of the 16S rRNA gene (Table S2). In contrast, all isolates grown on SI medium supplemented with 10% ethanol, which mimicked the environment of sake kasu, were identified as *F. fructivorans* (Table S2), suggesting that the isolated LAB were highly adapted to the environment of the orthodox narazuke manufacturing process. It was also predicted that this LAB was derived from the atmosphere in the two factories or from the sake kasu used in the previous batch because *F. fructivorans* was scarcely detected in salted vegetables and aged sake kasu (Fig. 2). The microbial compositions of narazuke from factories A, B, and C were quite different from those produced in factories N and M. In the final products from the factories A, B, and C, bacteria belonging to families Bacillaceae, Streptococcaceae, etc. dominated the microbiota, and *F. fructivorans* was not detected (Fig. 2), implying a significant impact of the orthodox manufacturing processes on the microbial dynamics in narazuke.

### Laboratory-scale narazuke fermentation test

**Fig 3:**
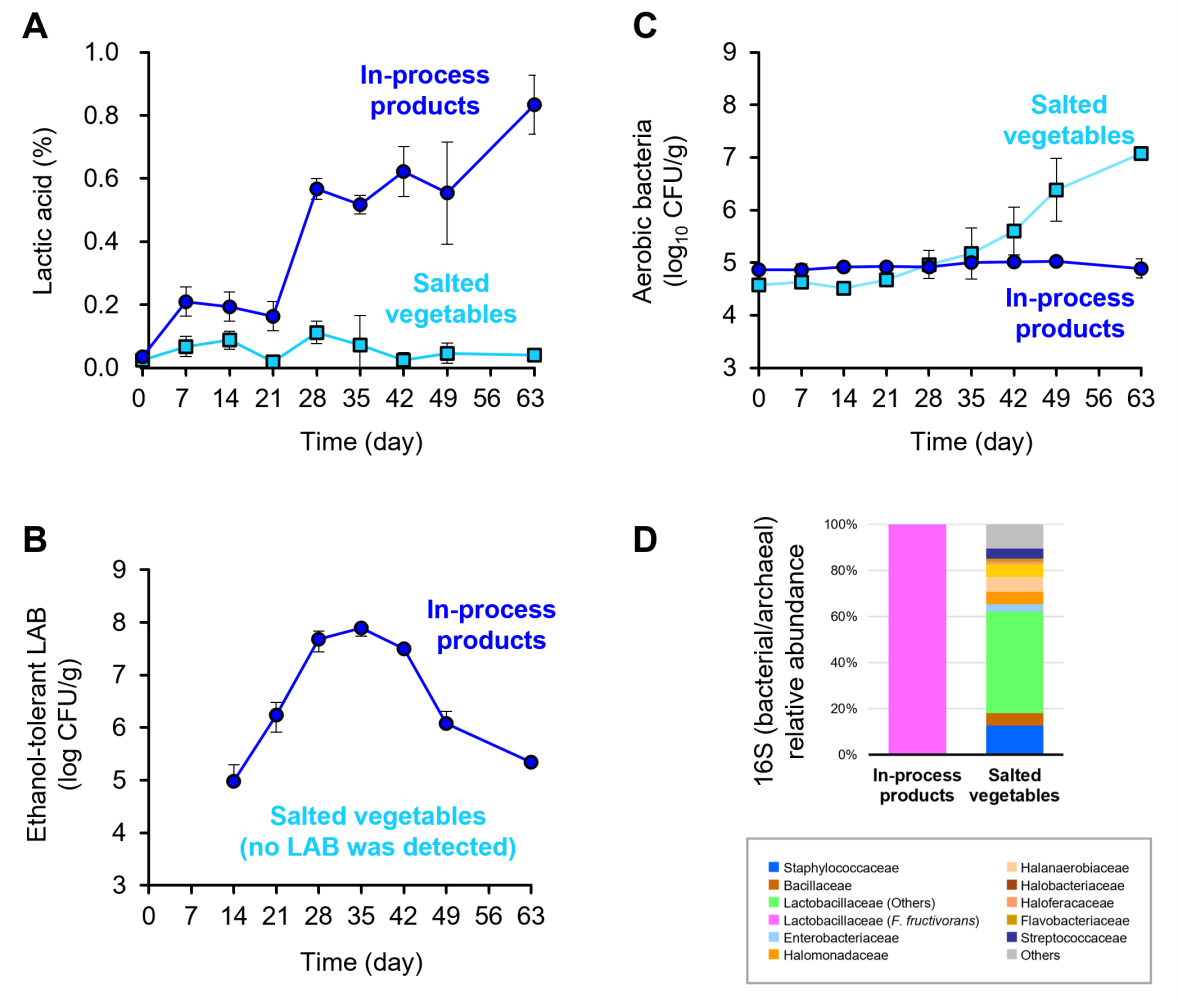
Laboratory-scale narazuke fermentation test. The in-process products collected from the early stage (embedding process #2) or salted vegetables before the embedding process were anaerobically fermented in aged sake kasu for 63 days. Lactic acid, viable cell numbers of ethanol-tolerant LAB, and those of aerobic bacteria in the samples were periodically monitored. Microbiota in the samples after 63 days of fermentation was also determined. (**A**) Lactic acid (%). (**B**) Ethanol-tolerant LAB (log_10_ CFU/g). (**C**) Aerobic bacteria (log_10_ CFU/g). (**D**) Microbiota in the laboratory-scale narazuke fermentation test 63 days after inoculation. The graph shows bacterial and archaeal 16S rRNA gene data at the family level, and the data represent the average values calculated from three independent experiments. (**A–C**) Experiments were conducted in triplicate, and error bars indicate standard deviations. Symbols: blue circles, in-process products; and light blue squares, salted vegetables.

Next, we performed a 2-month laboratory-scale fermentation test using the in-process product of gourd narazuke (collected from embedding process #2) and new aged sake kasu under anaerobic conditions, which reproduced embedding process #3 (Fig. 1). As a control, salted gourd before the embedding process was fermented in aged sake kasu. As indices of fermentation progression, we periodically monitored lactic acid production and the growth of ethanol-tolerant LAB using a colorimetric method and a drop-plate method using SI agar supplemented with 5% NaCl and 5% ethanol, respectively. When the in-process product was used, lactic acid sharply increased from 0.16% on day 21 to 0.57% on day 28, accompanied by gas generation (Fig. S1), and finally reached 0.83% by the end of fermentation (Fig. 3A). In parallel with the rapid increase in lactic acid at the middle stage of fermentation, the ethanol-tolerant LAB logarithmically proliferated from day 14 (8.7 × 10^4^ colony-forming units [CFUs]/g) to day 28 (4.8 × 10^7^ CFU/g), and the viable cell number of the LAB reached a maximum of 7.8 × 10^7^ CFU/g at day 35 (Fig. 3B). In contrast, the growth of other aerobic bacteria was static, and their viable cell numbers were maintained at ca. 1.0 × 10^5^ CFU/g throughout fermentation (Fig. 3C). 16S rRNA amplicon sequencing showed that the microbiota in the sample was dominated by LAB of the family Lactobacillaceae when the in-process products were fermented (Fig. 3D), and all isolates from the final product after 63 days of fermentation were identified as *F. fructivorans* (Table S3). *F. fructivorans* is a heterolactic acid bacterium that produces lactic acid, carbon dioxide, and ethanol as the major fermentation products (22), suggesting that the gas emitted during the fermentation test (Fig. S1) was carbon dioxide, which was attributed to heterolactic fermentation by the LAB. In the control test, the level of lactic acid never exceeded 0.2%, and no ethanol-tolerant LAB were detected throughout fermentation (Fig. 3A and B). Instead, other aerobic bacteria gradually increased from 4.7 × 10^4^ CFU/g on day 21 to 1.2 × 10^7^ CFU/g at the end of tests (Fig. 3C) with concomitant gas generation after day 49 (Fig. S1). Considering that lactic acid did not accumulate in the control tests, it was suggested that the gas was derived not from heterolactic fermentation but from other metabolic pathways. Amplicon sequencing analysis indicated that LAB, other than *F. fructivorans*, were the most dominant in the microbiota when salted gourds were used (Fig. 3D). These results suggested that lactic acid fermentation by *F. fructivorans*, which emerged from the in-process products at the early stage (embedding process #2) (Fig. 1), occurred in aged sake kasu.

To evaluate whether *F. fructivorans* affects the quality of narazuke, metabolites in narazuke samples obtained through laboratory-scale fermentation tests were comprehensively analyzed using capillary electrophoresis-Fourier transform mass spectrometry (CE-FTMS). The narazuke sample obtained after 63 days of fermentation using in-process products and aged sake kasu as ingredients (Fig. 3) contained significantly higher amounts of inosine, glutathione, and *S*-adenosylmethionine, all of which are known to be produced by LAB (and yeasts) involved in fermentation and largely contribute to the health-promoting effect of fermented foods (23–25), compared with the in-process products before embedding (day 0) (Table S4). These results suggest that *F. fructivorans* plays a key role in determining the distinct characteristics of narazuke.

### Impact of NaCl and various alcohols on the growth of *F. fructivorans* isolated from narazuke

**Fig 4:**
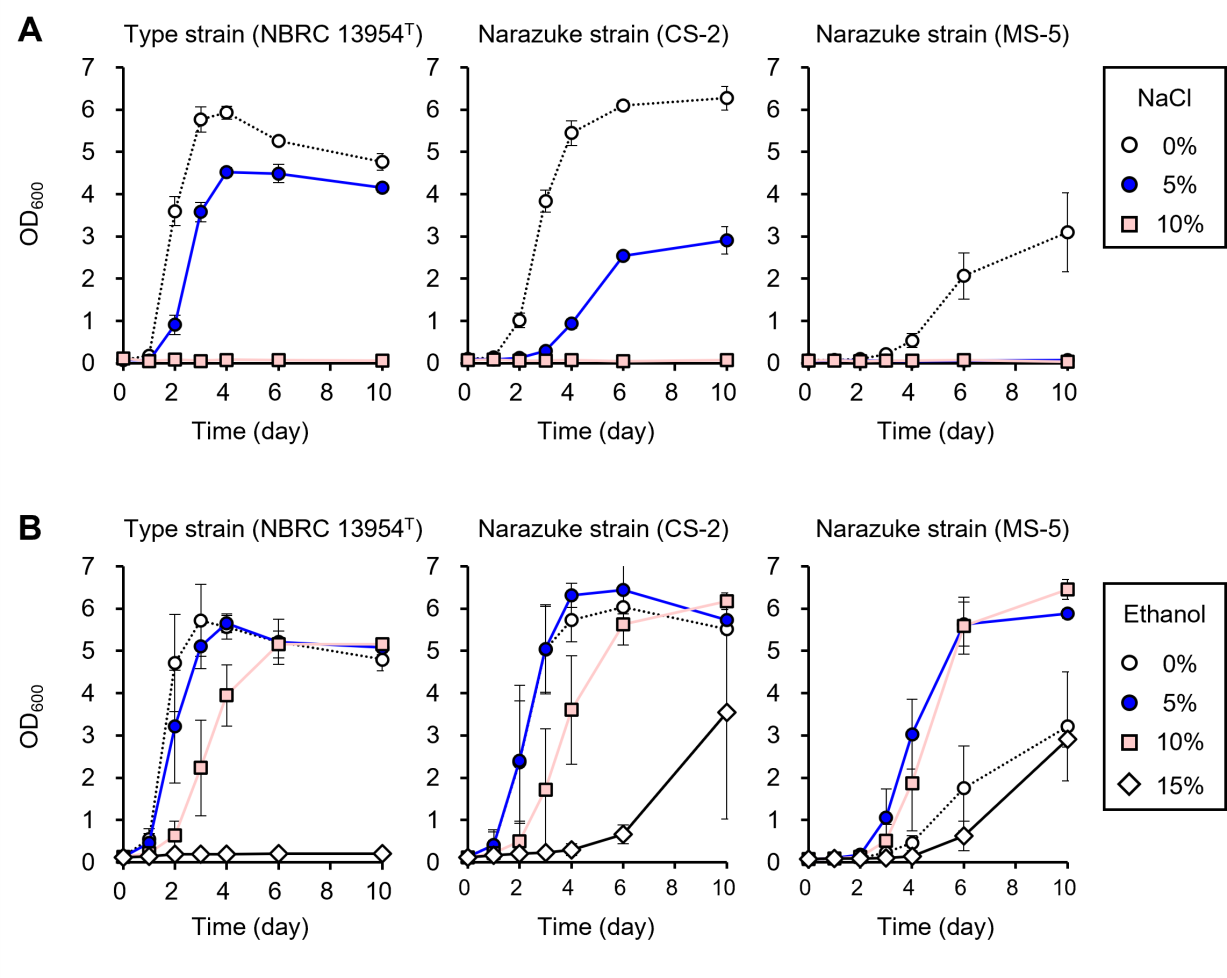
Effects of NaCl and ethanol on the growth of LAB strains isolated from narazuke. The narazuke strains *F. fructivorans* CS-2 and MS-5 were anaerobically cultivated in the presence of various concentrations of NaCl and ethanol, and their growth (OD_600_) was periodically monitored throughout cultivation. The type strain NBRC 13954^T^ was also subjected to the test as a reference. (**A**) Effect of NaCl on the growth of the type and the narazuke strains. Symbols: white circles, 0%; blue circles, 5%; and pink squares, 10%. (**B**) Effect of ethanol on the growth of the type and the narazuke strains. Symbols: white circles, 0%; blue circles, 5%; pink squares, 10%; and white diamonds, 15%. (**A and B**) Panels: left, NBRC 13954^T^; middle, CS-2; and right, MS-5. Experiments were conducted in triplicate, and error bars indicate standard deviations.

Microorganisms in narazuke-making environments are exposed to high concentrations of NaCl and ethanol. Considering that *F. fructivorans* dominated the microbial community in narazuke (Fig. 2 and 3D), it was suggested that this LAB was highly adapted to such harsh environments. To investigate this, we isolated two ethanol-tolerant LAB strains from the final products of factories N and M, both of which were identified as *F. fructivorans* based on the complete sequence of the 16S rRNA gene and designated as CS-2 and MS-5, respectively (Table 1). The two strains were cultivated in SI liquid medium containing various concentrations of NaCl, ethanol, methanol, or isopropanol. The type strain *F. fructivorans* NBRC 13954^T^ grew actively even in the presence of 5% NaCl (Fig. 4A) (Fig. S2). In contrast, CS-2 and MS-5 showed severe growth defects when cultivated in the presence of more than 5% NaCl (Fig. 4A) (Fig. S2). Other strains isolated from sake were also more sensitive to NaCl than the type strain (Table 1) (Fig. S2). These results indicated that the isolates from narazuke, as well as those from sake, were not as tolerant to high NaCl concentrations as the type strain. In contrast, the narazuke strains CS-2 and MS-5 exhibited high ethanol tolerance in the presence of 15% ethanol, whereas the growth of the type strain was inhibited in an ethanol concentration-dependent manner and was completely arrested in the presence of 15% ethanol (Fig. 4B) (Fig. S3). Furthermore, the isolated strains showed higher growth rates and final biomass in media containing 5%–10% ethanol than in ethanol-free medium (Fig. 4B) (Fig. S3), which is a unique characteristic known as ethanolphilicity (26). Similar high ethanol tolerance and ethanolphilicity were also observed in the sake strains (Fig. S3). In particular, MS-5 exhibited markedly potent ethanolphilicity, showing faster growth in media containing 5%–10% ethanol than in an ethanol-free medium (Fig. 4B) (Fig. S3). It should be noted that the growth of MS-5 was also remarkably enhanced in the presence of methanol and isopropanol (Fig. S4), indicating that ethanolphilicity is induced by not only ethanol but also other alcohols.

**TABLE 1:** *F*. *fructivorans* strains used in this study

Strain	Source or reference^*a*^
Narazuke strains	
CS-2^*b*^	This study
MS-5^*b*^	This study
Sake strains	
JCM 1198	JCM (26)
NBRC 13118	NBRC (26)
NBRC 15887	NBRC (26)
Other strains	
NBRC 13954^T^	NBRC
NBRC 14747	NBRC

^*a*^ JCM, Japan Collection of Microorganisms of the RIKEN BioResource Research Center (Japan); NBRC, NITE Biological Resource Center (Japan).

^*b*^ CS-2 and MS-5 were isolated from narazuke samples produced by factories N and M, respectively.

**Fig 5:**
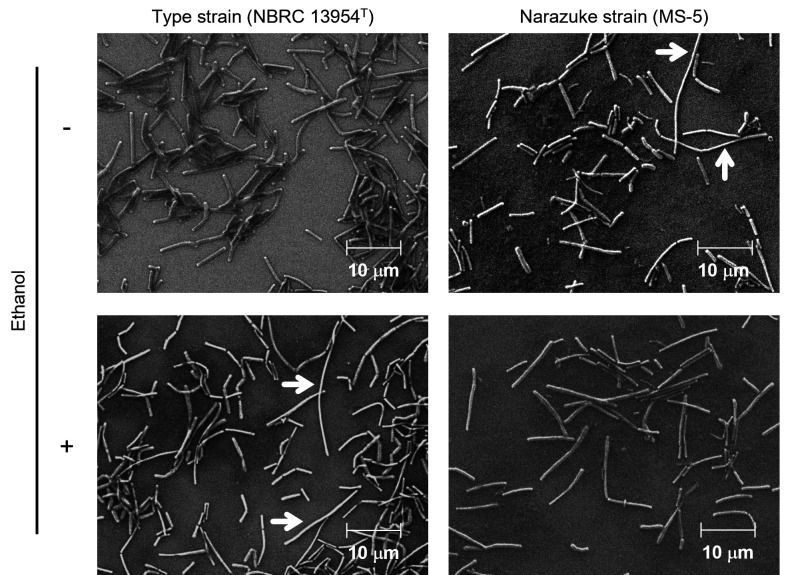
Scanning electron microscopic images of the cells of *F. fructivorans* NBRC 13954^T^ and the narazuke strain MS-5 in the logarithmic growth phase cultivated in SI medium lacking or containing 7.5% ethanol. Panels: left, NBRC 13954^T^; right, MS-5; upper, without ethanol; and lower, with ethanol. White arrows represent abnormally elongated cells. Scale bar, 10 µm.

To investigate the impact of ethanol on cell shape, we observed cells of NBRC 13954^T^ and MS-5 grown in SI medium lacking or containing ethanol using electron microscopy. When the type strain was cultivated in the absence of ethanol, the length of most cells was approximately 5–7 µm (10 µm >) (Fig. 5). In contrast, the narazuke strain exhibited an abnormally elongated cell morphology (>10 µm) compared with the type strain, and the length of some cells exceeded 20 µm (Fig. 5). We next cultivated the two strains in SI medium supplemented with ethanol. The results indicated that the anomalous phenotype of MS-5 observed in an ethanol-free environment was alleviated, whereas the cell length of the type strain was aberrantly elongated (Fig. 5). It has been well-known that environmental stresses such as high temperature, hydrostatic pressure, and ethanol content induce cell elongation by inhibiting cell division in LAB (27–29). These findings are well consistent with the impaired growth accompanied by the abnormal cell elongation of the type strain in the presence of ethanol (Fig. 4B and 5). In contrast, the narazuke strain exhibited the opposite responses to ethanol regarding cell growth and shape when compared with the type strain (Fig. 4B and 5), supporting ethanolphilicity of MS-5. Collectively, these findings suggest that high ethanol tolerance and ethanolphilicity are essential for the adaptation of narazuke strains to extreme environments during narazuke-making.

### Possible involvement of impaired fatty acid metabolism in ethanolphilicity

**Fig 6:**
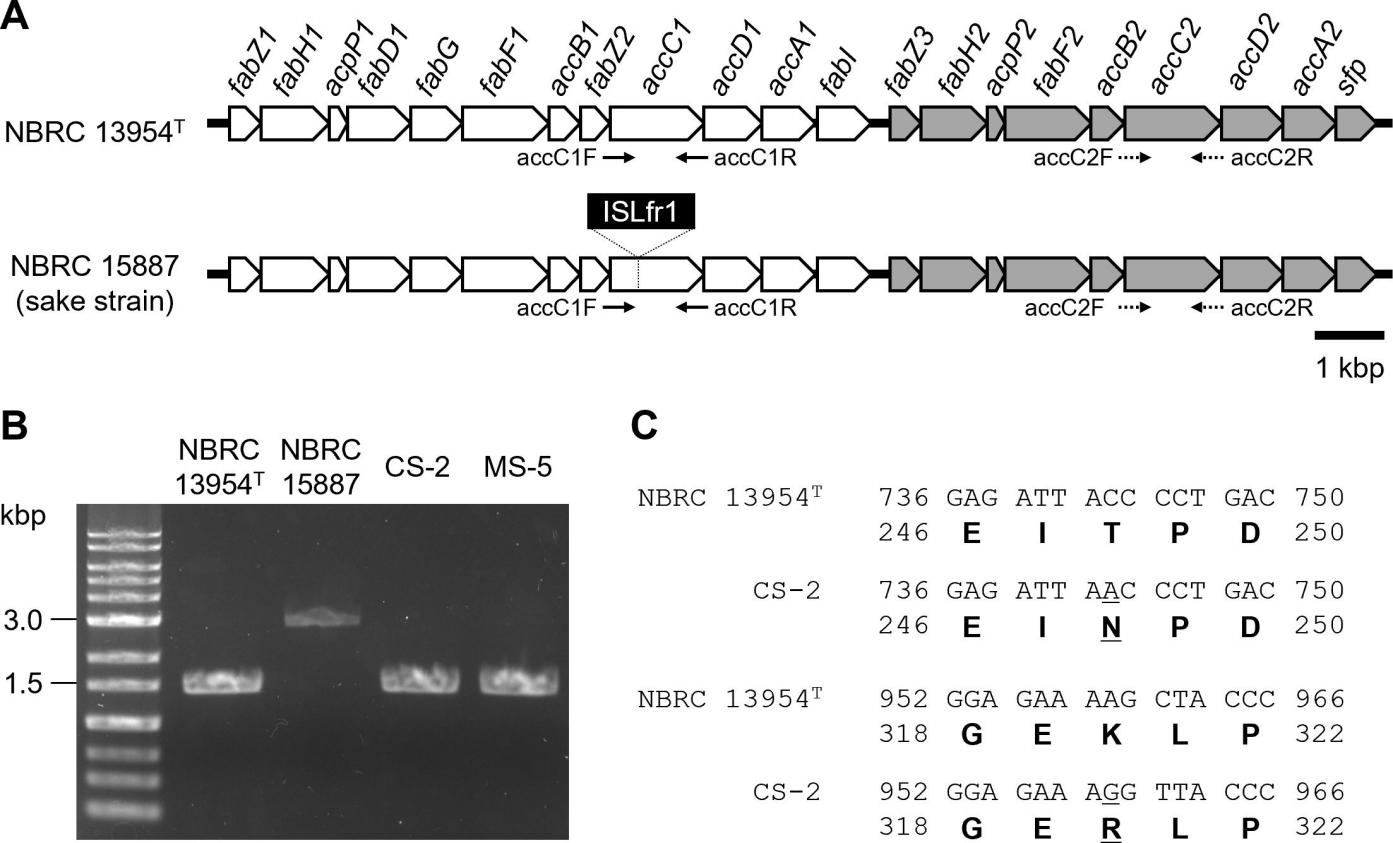
Genotyping of the *accC1* locus of various *F. fructivorans* strains. (**A**) Schematic representations of the gene clusters involved in fatty acid biosynthesis, including *accC* genes. The upper and lower diagrams correspond to the gene clusters of the type strain NBRC 13954^T^ and the sake strain NBRC 15887, respectively. Solid and dotted arrows indicate primers used for genotyping of the *accC1* (accC1F/accC1R) and *accC2* (accC2F/accC2R) genes, respectively. ISLfr1 represents a transposable element inserted into the *accC1* gene in the sake strain. The upstream and downstream gene clusters are colored white and gray, respectively. *fabZ*, 3-hydroxyacyl-acyl carrier protein (ACP) dehydratase; *fabH*, β-ketoacyl-ACP synthase III; *acpP*, ACP; *fabD*, ACP-S-malonyltransferase; *fabG*, 3-oxoacyl-ACP reductase; *fabF*, β-ketoacyl-ACP synthase II; *accB*, acetyl-CoA carboxylase biotin carboxyl carrier protein; *accC*, acetyl-CoA carboxylase biotin carboxylase subunit; *accD*, acetyl-CoA carboxylase carboxyltransferase subunit β; *accA*, carboxyltransferase subunit α; *fabI*, enoyl-ACP reductase; and *sfp*, 4′-phosphopantetheinyl transferase superfamily protein. (**B**) PCR analysis of the *accC1* locus. The *accC1* loci of NBRC 13954^T^, NBRC 15887, CS-2, and MS-5 were amplified from genomic DNA using the primer pair accC1F/accC1R shown in Fig. 6A. (**C**) Alignment of nucleotide and amino acid sequences of *accC1* between NBRC 13954^T^ and CS-2. Single-nucleotide polymorphisms accompanied by non-synonymous amino acid substitutions detected in CS-2 are underlined.

**Fig 7:**
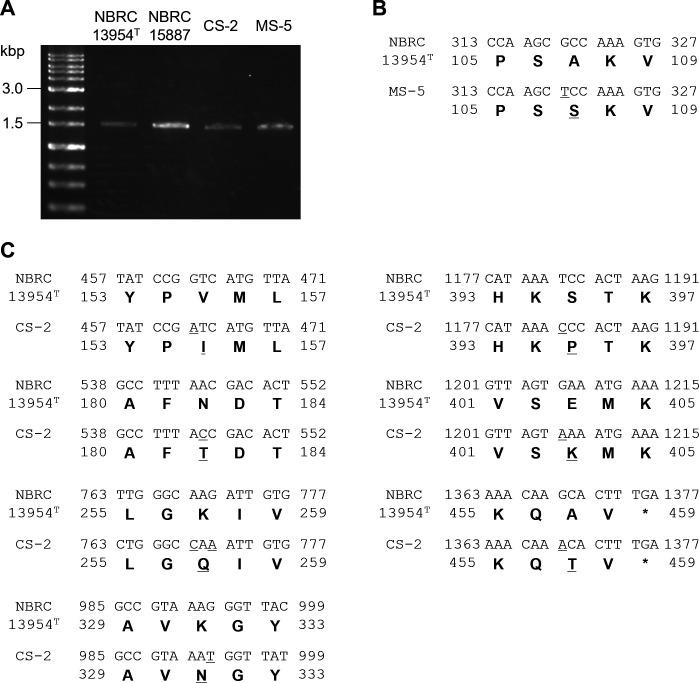
Genotyping of the *accC2* locus of various *F. fructivorans* strains. (**A**) PCR analysis of the *accC2* locus. The *accC2* loci of NBRC 13954^T^, NBRC 15887, CS-2, and MS-5 were amplified from genomic DNA using the primer pair accC2F/accC2R shown in Fig. 6A. (**B**) Alignment of nucleotide and amino acid sequences of *accC2* between NBRC 13954^T^ and MS-5. (**C**) Alignment of nucleotide and amino acid sequences of *accC2* between NBRC 13954^T^ and the CS-2. (**B and C**) Single-nucleotide polymorphisms accompanied by non-synonymous amino acid substitutions detected in the narazuke strains are underlined.

A previous study has reported that the *accC* gene, which encodes the biotin carboxylase subunit of acetyl-CoA carboxylase and is essential for fatty acid biosynthesis, is disrupted by the insertion of a transposon (ISLfr1) in the ethanolphilic *F. fructivorans* NBRC 15887, NBRC 13118, and JCM 1198 isolated from sake (26). This suggests the possible involvement of a loss-of-function mutation in *accC* in ethanolphilicity. Genome analysis revealed that the type strain NBRC 13954^T^ harbors two tandem gene clusters responsible for fatty acid biosynthesis (Fig. 6A, upstream and downstream clusters), and the two *accC* paralogs located in the upstream and downstream clusters were designated *accC1* and *accC2*, respectively (Fig. 6A). Genotypes of the *accC1* and *accC2* loci of the type, sake, and narazuke strains were determined using PCR with the primer pairs accC1F/accC1R and accC2F/accC2R (Fig. 6A), followed by sequencing of the PCR products. For *accC1*, the target locus of the sake strain NBRC 15887 was longer than that of the type strain (Fig. 6B), and DNA sequencing confirmed the insertion of ISLfr1 into the *accC1* locus of the sake strain, which is consistent with the previous description (26). However, contrary to expectations, the length of the *accC1* locus from the narazuke strains CS-2 or MS-5 was the same as that of the type strain (Fig. 6B). Although non-synonymous nucleotide substitutions (T248N and K320R) were detected in *accC1* of CS-2 (Fig. 6C), the sequence of the target locus in MS-5 was 100% identical to that of the type strain, and no apparent mutation leading to loss-of-function mutation of the gene (e.g., premature termination, deletion, or insertion causing frameshift) was observed in the narazuke strains. Similar tendencies were also observed in the *accC2* loci of the narazuke strains; the length of *accC2* loci was the same among the four strains (Fig. 7A) and no insertion or deletion culminating in a frameshift followed by premature termination was observed in the two narazuke strains, though several non-synonymous substitutions were found when compared with the nucleotide sequence of *accC2* in the type strain (Fig. 7B and C). The sequence of the *accC2* gene in the sake strain NBRC 15887 was identical to that of the type strain (data not shown). Whereas the non-synonymous nucleotide substitutions detected in *accC1* (Fig. 6C) and *accC2* (Fig. 7B and C) of the narazuke strains might negatively affect AccC functions, these findings suggested that the ethanolphilicity of the narazuke strains was attributed not only to the loss-of-function of the *accC* genes but also to unidentified factors.

**Fig 8:**
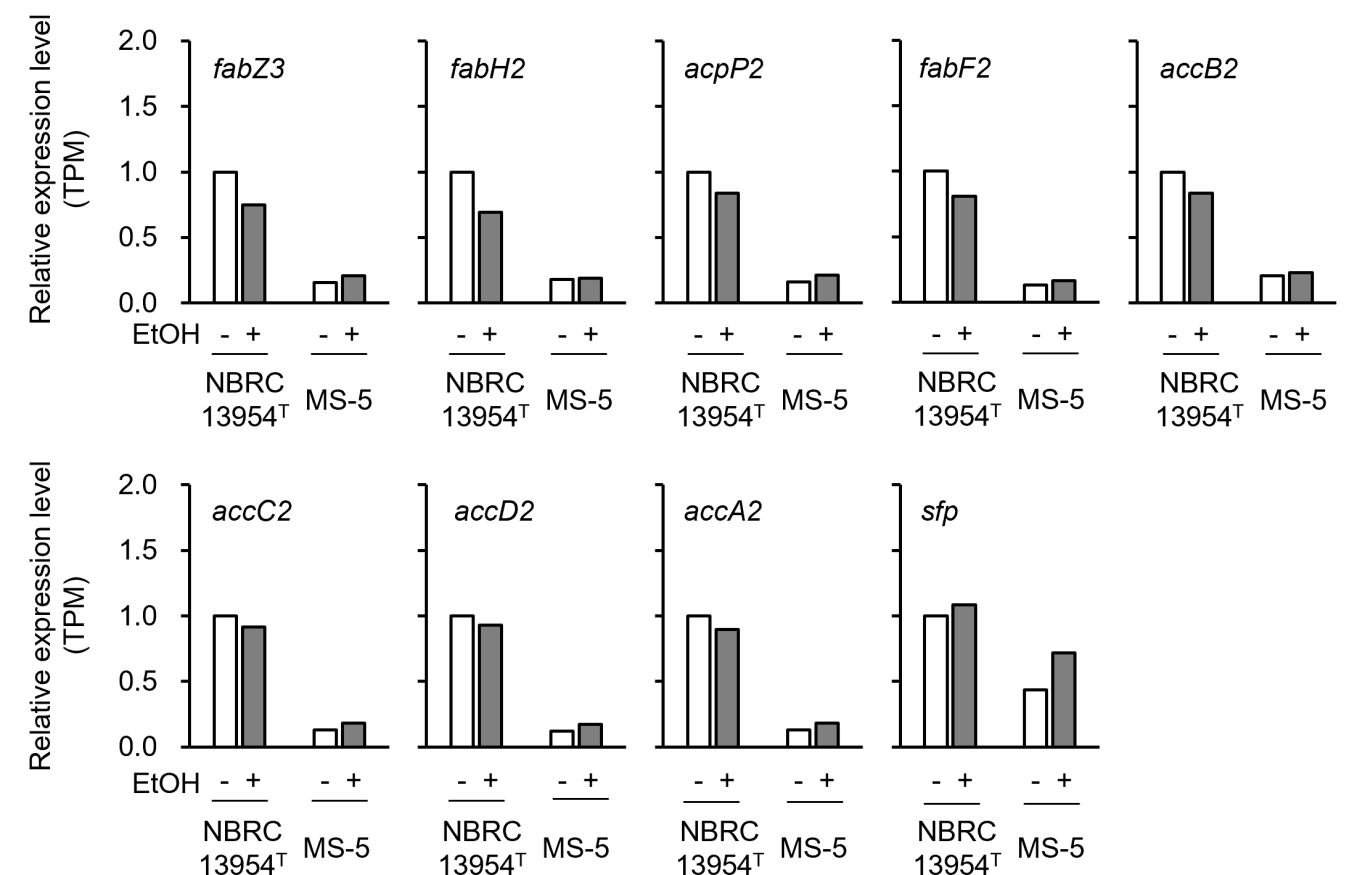
Relative expression levels of genes constituting the downstream cluster responsible for fatty acid biosynthesis in the narazuke strain MS-5. The type strain NBRC 13954^T^ and the narazuke strain MS-5 were cultivated in SI medium supplemented with or without ethanol until the cultures reached the late logarithmic growth phase. Total RNA extracted from the cells was then subjected to mRNA-sequencing as described in the Materials and Methods section. The expression level of each gene was normalized to that of the type strain grown in the absence of ethanol, which was set to 1. Bars: white, without ethanol; and gray, with ethanol. See the legend to Fig. 6 for a description of each gene. TPM, transcripts per million reads.

To examine this, we focused on the narazuke strain MS-5, which exhibited more potent ethanolphilicity than CS-2 (Fig. 3B), and performed a comparative transcriptomic analysis (messenger RNA [mRNA]-sequencing) using total RNA extracted from cells of the type strain and MS-5 cultivated in SI medium containing or lacking 7.5% ethanol. A total of 235 and 411 differentially expressed genes (DEGs) were identified between the type and narazuke strains in the presence and absence of ethanol, respectively. We further screened the genes whose expression levels were significantly changed specifically in MS-5, showing that the genes comprising the downstream cluster for fatty acid biosynthesis (*fabZ3–accA2*, except for *sfp*) (Fig. 6A) were remarkably downregulated (log_2_ [fold change <FC > ] < −1) in MS-5 regardless of the presence or absence of ethanol (Fig. 8). In contrast, no significant differences were found in the expression levels of genes constituting the upstream cluster under any of the conditions tested (Fig. S5). Together with the fact that the sake strain NBRC 15887, possessing the loss-of-function mutation in *accC1*, exhibits ethanolphilicity, these results again suggested that altered fatty acid synthesis caused by gene disruption or transcriptional downregulation in the fatty acid biosynthetic gene cluster (Fig. 6A and 8) leads to the acquisition of ethanolphilicity.

## DISCUSSION

Amplicon sequencing analysis targeting fungal ITS1 showed that microbes belonging to the genera *Aspergillus* and *Saccharomyces* dominated the microbiome in new and aged sake kasu, in-process, and final products (Fig. 2). Sake is produced using *Aspergillus oryzae* for the degradation of rice starch to glucose, and *S. cerevisiae* for alcoholic fermentation (Fig. 1) (30). As such, it would be valid that the dominant fungi in sake kasu were *A. oryzae* and *S. cerevisiae*, the latter of which was isolated from fresh sake kasu (Table S1). In salted vegetables, the microbiomes were dominated by yeasts of the genus *Zygosaccharomyces* (Fig. 2), and almost all isolates were identified as *Z. rouxii* as expected (Table S1). *Z. rouxii* is a major microorganism that plays a key role in determining the quality of traditional Japanese fermented foods with high salt content, such as soy sauce and soybean paste, because it produces various organic acids and volatile compounds (e.g., ethanol and esters) (18, 19). Yeasts belonging to the genera *Yamadazyma*, *Wickerhamiella*, and *Wickerhamomyces* were detected in salted vegetables and in-process products (Fig. 2), whereas *W. subpelliculosus* and *W. versatilis* were isolated from salted vegetables (Table S1). These halotolerant yeasts are reported to contribute to the development of pleasant aromas in soy sauce by producing diverse organic volatile compounds during aging (21), suggesting their significance in the quality of salted vegetables.

16S rRNA amplicon sequencing revealed a similar tendency in the succession of microbial communities in the samples collected from factories N and M that produced narazuke using the orthodox method. Bacteria in the families Staphylococcaceae and Bacillaceae and various halotolerant bacteria and archaea were abundant in aged sake kasu and salted vegetables, respectively (Fig. 2). In contrast, *F. fructivorans* dominated the narazuke microbiota throughout the manufacturing process, except for the embedding process #3 (Fig. 2). The laboratory-scale fermentation tests that mimicked the embedding process #3 also culminated in the occupation of the final product niche by *F. fructivorans* accompanied by an increase in lactic acid concentration and viable cell number of ethanol-tolerant LAB (Fig. 3). In contrast, the microbial community at the end of fermentation in the control test using salted vegetables was dominated by bacteria of the family Lactobacillaceae, other than *F. fructivorans* (Fig. 3). During the production of fermented vegetables, the growth of aerobic bacteria is generally inhibited by the metabolites of LAB involved in fermentation, resulting in enhanced preservability (1). Together with the fact that no growth of aerobic bacteria was observed when the in-process products were fermented in aged sake kasu (Fig. 3), these findings strongly suggest that *F. fructivorans* is the LAB responsible for narazuke fermentation. The decrease in the relative abundance of *F. fructivorans* in the embedding process #3 was recovered in the final product (Fig. 1). In narazuke-making, aged sake kasu used in the embedding processes #3 and #2 is reused in the former embedding steps #2 and #1, respectively (Fig. 1), which closely resembles backslopping (31). During the embedding process #3, in-process products from the process #2, whose niche was occupied by *F. fructivorans*, were embedded in new aged sake kasu dominated by other bacteria (Fig. 2). As demonstrated in the laboratory-scale fermentation tests (Fig. 3), *F. fructivorans* preferentially grew and dominated the microbial community in the narazuke production environment. Thus, it is considered that the orthodox manufacturing process enables stable and systematic narazuke production by repeated inoculation of *F. fructivorans* via backslopping. In contrast, the relative abundance of *F. fructivorans* was significantly lower (1% >) in the final products from factories A, B, and C than in those from factories N and M. Bacteria in the families Bacillaceae, Enterobacteriaceae, and Streptococcaceae were dominant in the former (Fig. 2). These results suggest that manufacturing processes have a significant impact on the development of the narazuke microbiome and eventually affect the quality of the final products.

In general, microorganisms from bacteria (*Zymomonas mobilis*, *Escherichia coli*, etc.) to yeasts (*Kluyveromyces marxianus*, *S. cerevisiae*, etc.) decrease the fluidity and permeability of cell membranes by increasing the ratio of unsaturated and *trans*-monoenoic fatty acids in membrane lipids in response to ethanol, leading to the acquisition of ethanol tolerance through elevated membrane hydrophobicity (32). This generality of ethanol tolerance implies that *F. fructivorans* has a similar mechanism for resisting ethanol toxicity. It is well-known that tolerance to ethanol is markedly higher in *F. fructivorans* than in other bacteria (13). Consistent with this, the type strain NBRC 13954^T^ exhibited relatively active growth, even in the presence of 10% ethanol (Fig. 4B). It is of interest that the type strain harbored two tandemly arranged fatty acid biosynthetic gene clusters in its genome (Fig. 6A), probably due to gene duplication. To the best of our knowledge, other bacteria, including *Streptococcus pneumoniae* (33) and *E. coli* (34), possess a single gene cluster and a set of genes, respectively, involved in fatty acid biosynthesis, and the disruption of any gene involved in fatty acid synthesis is lethal in these bacteria (35, 36). Enhanced fatty acid biosynthesis caused by the duplication of related gene clusters may potentiate ethanol tolerance in *F. fructivorans* by increasing cell membrane hydrophobicity. At the same time, the current study suggests that ethanolphilicity is linked to the loss of function in one of these gene clusters (i.e., *accC1* disruption in the sake strains [Fig. 6A and B] or lowered expression of genes comprising the downstream cluster in the narazuke strain [Fig. 8]). Considering that the disruption of genes responsible for fatty acid synthesis results in lethality in other bacteria (35, 36), it appears contradictory that the dysfunction in one of the two clusters provokes ethanolphilicity, but not cell death, in *F. fructivorans*. The redundancy in fatty acid biosynthesis may prevent the lethality ascribed to partial loss-of-function in either of the two clusters while causing an imbalance in fatty acid metabolism. This may lead to changes in membrane properties and eventually affect ethanolphilicity (see below).

Comparative transcriptomic analysis revealed that the expression levels of genes comprising the downstream gene cluster for fatty acid biosynthesis (Fig. 6A) were significantly lower in the narazuke strain MS-5 than in the type strain (Fig. 8). All the ethanolphilic sake strains used in this study (NBRC 15887, NBRC 13118, and JCM 1198) (Table 1) had a loss-of-function mutation in the *accC1* gene (26). It is noteworthy that the narazuke strain MS-5 showed markedly poorer growth in ethanol-free medium than in media containing 5%–10% ethanol (Fig. 4B) (Fig. S3). This compromised growth was also supported by the abnormal cell division of MS-5 in the absence of ethanol (Fig. 5). Previous studies have reported that ethanolphilic sake strains harboring loss-of-function mutations in *accC1* produce unusual fatty acids with extremely long carbon chains (C_22_–C_30_) through unknown mechanisms (37, 38). These findings suggest that very long-chain fatty acids with elevated hydrophobicity are synthesized through altered fatty acid metabolism in narazuke and sake strains. Such metabolic perturbations would make the cell membrane more rigid and eventually culminate in a decrease in cell adaptability to the ethanol-free environment by lowering the membrane fluidity and permeability with concomitant inhibition of enzymes/proteins working on the cell surface and within the membrane. In contrast, while ethanol is deleterious for microbial survival, it increases the permeability of the cell membrane by lowering its dielectric strength, which allows this primary cell barrier to accommodate charged/polar molecules (39). Alcohols and solvents with a lower polarity than water are also known to decrease cell membrane hydrophobicity (39). Therefore, ethanol, methanol, and isopropanol (Fig. S4) were predicted to alleviate the anomalous rigidity of cell membranes caused by very long-chain fatty acids in ethanolphilic *F. fructivorans*. This may explain why the impaired growth of the ethanolphilic strain MS-5 in alcohol-free environments was recovered by the addition of various alcohols. Further studies are required to elucidate the molecular mechanisms underlying the effects of ethanolphilicity.

This is the first report to identify a unique LAB with ethanolphilicity from the traditional Japanese preserved (fermented) food narazuke. Our findings suggest that the ethanolphilicity is key to adapting *F. fructivorans* to harsh environments for narazuke-making. We are currently investigating the molecular mechanisms of the ethanolphilicity in the LAB strains. These findings are expected to be applicable to the development of robust industrial microbes with high adaptability to alcohols and novel enzymes that function in the presence of ethanol and solvents.

## MATERIALS AND METHODS

### Amplicon sequencing analysis of narazuke samples

The narazuke samples used in this study (ingredients [salted vegetables and sake kasu], in-process products, final products, and saturated salt water for soaking vegetables) were provided by Naraya Honten (Factory N, Nara, Japan) and Morinaraduketen Co., Ltd. (Factory M, Nara, Japan). The commercially available final products manufactured by Factory A, B, and C were also used as references. The in-process products in the early and late stages correspond to the samples during embedding processes #2 and #3, respectively, as shown in Fig. 1. DNA extraction from the samples and amplicon sequencing analyses were conducted by Bioengineering Lab Co., Ltd. (Sagamihara, Japan), in accordance with a previously reported method (40, 41). The ITS1 and V4 regions of the 16S rRNA gene were PCR-amplified using the primer pairs ITS1F-KYO1/ITS2-KYO2 and 515f/806r, respectively (Table S5). Sequencing was conducted using a paired-end, 2 × 300 bp cycle run on a MiSeq sequencing system and MiSeq Reagent Kit version 3 (Illumina, San Diego, CA, USA). After quality filtering and chimera checking of the sequencing reads, operational taxonomic units (OTUs) were predicted using QIIME2. OTUs accounting for more than 1% of the total sequence reads were classified, while taxa with an abundance of less than 1% were grouped as “others” using the EzBioCloud 16S rRNA database ver. 20210707 (42).

### Isolation and identification of microorganisms from narazuke samples

General DNA manipulations were performed as previously described by Green and Sambrook (43). To isolate eukaryotic microbes, 2 g of each sample was suspended in 15 mL of yeast-peptone-dextrose (YPD) medium (1% yeast extract [Thermo Fisher Scientific Inc., Waltham, MA, USA], 2% casein peptone [Nacalai tesque Inc., Kyoto, Japan], and 2% glucose) supplemented with 100 µg/mL chloramphenicol to inhibit bacterial growth and statically incubated at 30°C for 3 days. An aliquot (100 µL) of the diluted culture was then spread on YPD agar plates containing 100 µg/mL chloramphenicol and further incubated at 30°C for 3 days. Single colonies grown on the plates were isolated by repeated streaking on YPD agar plates containing chloramphenicol. The isolates were identified by sequencing the ITS1 region of the rRNA gene, which was PCR-amplified using the primer pair ITS_1F/ITS_1R (Table S5) and using genomic DNA as a template.

As for LAB isolation, MRS broth (Biokar Diagnostics, Paris La Défense Cedex, France) supplemented with 50 µg/mL sodium azide and 50 µg/mL cycloheximide was used to inhibit the growth of aerobic and eukaryotic microorganisms. Each narazuke sample (2 g) was suspended in 15 mL of MRS containing the two antibiotics and anaerobically cultivated at 30°C for 3 days using the AnaeroPack system (Mitsubishi Gas Chemical, Tokyo, Japan), which generates carbon dioxide while simultaneously absorbing oxygen to create an anaerobic atmosphere. An aliquot (100 µL) of the diluted culture was then spread on MRS agar plates supplemented with 50 µg/mL sodium azide, 50 µg/mL cycloheximide, and 0.5% CaCO_3_ and anaerobically incubated at 30°C for 3 days. Single colonies grown on the plates were isolated by repeated streaking onto MRS agar plates. To isolate ethanol-tolerant LAB, 2 g of each sample was suspended in 15 mL of SI medium (1% yeast extract, 0.5% polypeptone, 2.5% glucose, 0.01% MgSO_4_･7H_2_O, 0.00025% MnSO_4_･4–6H_2_O, 0.00025% FeSO_4_･7H_2_O, 1% sodium acetate, 0.0005% mevalonic acid, 50 µg/mL sodium azide, and 0.06% agar, pH 5.0) (The Brewing Society of Japan, Tokyo, Japan) containing 10% ethanol and anaerobically incubated at 30°C for 7 days. An aliquot (100 µL) of the diluted culture was spread on SI agar plate containing 10% ethanol and further anaerobically cultivated at 30°C for 7 days. Single colonies were isolated by repeated streaking onto SI agar plates containing ethanol. The isolates were identified by sequencing the V4 regions of the 16S rRNA gene, which was PCR-amplified using the primer pair 515f/PC3mod (Table S5) and genomic DNA as a template.

### Laboratory-scale narazuke fermentation test

For the fermentation tests, we used ingredients (in-process products, salted vegetables, and aged sake kasu) provided by factory N. Salted vegetables were prepared by soaking fresh vegetables in saturated salt water for 2 months in factory N. Twenty grams of the in-process products collected from embedding process #2 or salted vegetables before the embedding process (Fig. 1), and 20 g of aged sake kasu were mixed in a 50 mL tube. Sake kasu stuck to the inner walls of the tube was dropped to the bottom of the tube through centrifugation (2,000 × *g*, 1 min). The mixtures were anaerobically cultured using the AnaeroPack system (Mitsubishi Gas Chemical) at 25°C for 63 days with stirring every 7 days. The sake kasu portion of the mixture was periodically sampled for subsequent analysis.

To quantify lactic acid, 0.1 g of each sample was suspended in 1 mL of sterilized water, and the supernatant of the suspension was collected through centrifugation (10,000 × *g*, 1 min). The pH of the resultant supernatant was adjusted to pH 8.0 with NaOH, and the lactic acid concentration was determined using Enzytec Liquid D-/L-Lactic acid (R-Biopharm AG, Darmstadt, Germany).

Viable cell counts of ethanol-tolerant LAB in the samples were determined using the drop-plate method. Briefly, 0.05 g of the sample was suspended in 900 µL of 5% NaCl. Ten-fold serial dilutions of the suspension were prepared, and 10 µL of each dilution was spotted five times onto SI agar plates supplemented with 5% NaCl and 5% ethanol. After anaerobic incubation at 30°C for 14 days, the colonies grown on the plate were counted. To measure the number of viable aerobic (general) bacteria, the common pour-plate method was used; 0.05 g of each sample was suspended in 900 µL of saline (0.85% NaCl). Similarly, 10-fold serial dilution of the suspension was prepared, and 100 µL of each dilution was spread on a plate count agar (PCA) agar plate (0.25% yeast extract [Thermo Fisher Scientific Inc.], 2% casein peptone [Nacalai Tesque Inc.], 0.1% glucose, and 1.5% agar). Colonies grown on the plate after three days of incubation at 30°C were counted as the number of viable bacteria.

The microbial communities of the samples obtained after 63 days of fermentation were also investigated using 16S rRNA amplicon sequencing, similarly to those of the narazuke samples (see above).

### Metabolomic analysis

The narazuke sample obtained from the laboratory-scale fermentation test (see above) was suspended in 500 µL of methanol containing 2 µM internal standards (H3304-1002, Human Metabolome Technologies Inc. [HMT], Tsuruoka, Japan) and homogenized using a homogenizer. Ultrapure water (500 µL) was then added to the homogenate, and the mixture was centrifuged (4°C, 2,300 × *g*, 5 min) to recover the supernatant. The resultant supernatant was filtered through the Ultrafree-MC centrifugal filter unit (HMT) with a 5 kDa cutoff (Millipore Sigma, Burlington, MA, USA) at 9,100 × *g* at 4°C to remove macromolecules. The flow-through-containing analytes were evaporated and reconstituted with the appropriate volume of ultrapure water. The metabolites in the samples were analyzed using the ω Scan package (HMT) with CE-FTMS using the Agilent CE system (Agilent Technologies Inc., Santa Clara, CA, USA) equipped with Q Exactive Plus (Thermo Fisher Scientific Inc.). The systems were connected by a fused silica capillary (50 µm inner diameter × 80 cm total length). H3301-1001 (HMT) and I3302-1023 (HMT) electrophoresis buffers served as electrolytes for cation and anion analyses, respectively. The spectrometer scanned samples from *m*/*z* 60 to 900 in the positive mode, and from *m*/*z* 70 to 1,050 in the negative mode, respectively (44). Peaks with a signal-to-noise ratio of >3 were automatically extracted using MasterHands automatic integration software ver.2.19.0.2 (Keio University, Tsuruoka, Japan) to obtain the data about *m*/*z*, peak areas, and migration times (45). Peaks corresponding to the adduct and fragment ions were excluded, and the remaining peaks were annotated in accordance with the HMT metabolite database based on their *m*/*z* values and migration times. The areas of the annotated peaks were normalized to the internal standards and sample volumes to determine the relative levels of each metabolite.

### LAB growth test

The *F. fructivorans* used in the growth tests are listed in Table 1. All strains, except for those isolated from narazuke (CS-2 and MS-5), were obtained from the NITE Biological Resource Center (NBRC, Kisarazu, Japan) and the Japan Collection of Microorganisms (JCM) of the RIKEN BioResource Research Center (Tsukuba, Japan). Each strain was anaerobically pre-cultured in 4 mL of SI medium at 30°C. An aliquot of the pre-culture was inoculated to 4 mL of SI medium supplemented with various concentrations of NaCl (0, 2.5, 5, 7.5, 10, 12.5, or 15%), ethanol (0, 2.5, 5, 7.5, 10, 12.5, 15, 17.5, or 20%), methanol (0, 2.5, 5, 7.5, 10, 12.5, 15, 17.5, or 20%), or isopropanol (0, 2.5, 5, 7.5, 10, 12.5, 15, 17.5, or 20%), such that an initial cell density (optical density at a wavelength of 600 nm [OD_600_]) of 0.1 was achieved. The cells were then anaerobically cultivated at 30°C for 10 days, and the OD_600_ values were periodically monitored.

### Scanning electron microscopy

*F. fructivorans* NBRC 13954^T^ and the narazuke strain MS-5 were pre-cultured as described above. An aliquot of the pre-culture was inoculated to 2 mL of SI medium supplemented with or without 7.5% ethanol to adjust the initial OD_600_ value to 0.1, and the two strains were anaerobically cultivated at 30°C for 3 days. Cells in the late logarithmic growth phase were collected from 1 mL of culture through centrifugation (4°C, 10,000 × *g*, 2 min), and the resultant pellet was mixed with 1 mL of 0.1 M phosphate buffer (pH 6.0) containing 1% glutaraldehyde, followed by incubation at room temperature for 2 h. The suspension was again centrifuged, and the collected cells were washed three times with 1 mL of 0.1 M phosphate buffer (pH 6.0). After discarding the supernatant, the cells were observed using the Miniscope TM4000 Plus II tabletop scanning electron microscope (Hitachi, Tokyo, Japan). Accelerating voltage of the microscope and magnification were set to 5 kV and 1,000 ×, respectively.

### Genotyping of the *accC* loci

The *accC1* and *accC2* loci of the type strain NBRC 13954^T^, sake strain NBRC 15887, and narazuke strains CS-2 and MS-5 were PCR-amplified from genomic DNA using accC1F/accC1R and accC2F/accC2R primer pairs, respectively (Fig. 6A) (Table S5). The resulting PCR products were then sequenced.

### mRNA-sequencing

*F. fructivorans* NBRC 13954^T^ and narazuke strain MS-5 were pre-cultured as described above. An aliquot of the pre-culture was passaged into 4 mL of SI medium containing or lacking 7.5% ethanol, and the cells were anaerobically cultivated as in the LAB growth tests (see above) until the cultures reached the late logarithmic growth phase. Total RNA from each strain was extracted using the RNeasy Mini Kit (Qiagen, Hilden, Germany) according to the manufacturer’s instructions. The obtained RNA samples were sent to Rhelixa Inc. (Tokyo, Japan) and sequenced using Illumina NovaSeq X Plus (Illumina) with a paired-end sequencing length of 150 bp for 1 G bases per sample, using strand-specific RNA-seq with Ribo-Zero Plus rRNA Depletion Kit (Illumina) and NEBNext Ultra II Directional RNA Library Prep Kit (New England Biolabs Inc., Ipswich, MA, USA). The quality of the raw paired-end sequence reads was evaluated using FastQC ver.0.11.7 (https://www.bioinformatics.babraham.ac.uk/projects/fastqc/). Sequence reads with low quality (< 20 b) and adapter sequences were trimmed using Trimmomatic software ver.0.38 with the following parameters: ILLUMINACLIP: path/to/adapter. fa:2:30:10, LEADING:20, TRAILING:20, SLIDING WINDOW:4:15, and MINLEN:36. The trimmed reads were then aligned to the reference genome (*F. fructivorans* type strain, NBRC 13957^T^, ASM336839v1 [GCF_003368395.1] [https://www.ncbi.nlm.nih.gov/datasets/genome/GCF_003368395.1/]) using RNA-seq aligner HISAT2 ver.2.1.0. Raw read counts were normalized with transcripts per million reads (TPM). Relative TPM values of each gene were normalized to those of the type strain NBRC 13954^T^ cultivated in SI medium lacking ethanol, which were set to 1. DEGs were detected with a threshold of |log_2_ (FC)| > 1.

## Data Availability

All sequences determined in the metagenomic analysis were deposited in the DNA Data Bank of Japan (DDBJ) under the accession numbers PRJDB19207, PRJDB19208, and PRJDB19209. mRNA-sequencing data were deposited in DDBJ under the accession number PRJDB35876. The 16S rRNA gene sequences of narazuke strains CS-2 and MS-5 were deposited in DDBJ under the accession numbers LC848309 and LC848310, respectively. The accC1 sequences of CS-2 and MS-5 were deposited in DDBJ under accession numbers LC848311 and LC848312, respectively. The accC2 sequences of CS-2 and MS-5 were deposited in DDBJ under accession numbers LC886233 and LC886234, respectively.

## SUPPLEMENTAL MATERIAL

Figures S1 to S5 aem.01730-25-s0001.pdf Fig. S1: Gas generation in the laboratory-scale narazuke fermentation test. Fig. S2: Effect of NaCl on growth of various *F. fructivorans* strains. Fig. S3: Effect of ethanol (EtOH) on growth of various *F. fructivorans* strains. Fig. S4: Effect of methanol (MeOH) and isopropanol (IPA) on growth of *F. fructivorans* strains. Fig. S5: Relative expression levels of genes constituting the upstream cluster responsible for fatty acid biosynthesis in the narazuke strain MS-5.

Tables S1 to S5 aem.01730-25-s0002.pdf Table S1: The preliminary identification of yeasts isolated from the narazuke samples based on ITS1 sequencing. Table S2: The preliminary identification of bacteria isolated from the narazuke samples based on 16S rRNA gene sequencing. Table S3: The preliminary identification of bacteria isolated in the laboratory-scale narazuke fermentation test (at day 63 after inoculation) based on 16S rRNA gene sequencing. Table S4: The compounds specifically detected in the samples after 63 days of the laboratory-scale narazuke fermentation test. Table S5: Primers used in this study.

ASM does not own the copyrights to Supplemental Material that may be linked to, or accessed through, an article. The authors have granted ASM a non-exclusive, world-wide license to publish the Supplemental Material files. Please contact the corresponding author directly for reuse.
