# Supplementary material for: Ethanolphilic lactic acid bacterium Fructilactobacillus fructivorans as the key microorganism for fermentation of narazuke, a traditional Japanese preserved food
Source: Appl Environ Microbiol. 2025 Nov 18;91(12):e01730-25. doi: 10.1128/aem.01730-25 (PMC12724224; doi:10.1128/aem.01730-25)
Supplement: Figures S1 to S5 — Fig. S1: Gas generation in the laboratory-scale narazuke fermentation test. Fig. S2: Effect of NaCl on growth of various F. fructivorans strains. Fig. S3: Effect of ethanol (EtOH) on growth of various F. fructivorans strains. Fig. S4: Effect of methanol (MeOH) and isopropanol (IPA) on growth of F. fructivorans strains. Fig. S5: Relative expression levels of genes constituting the upstream cluster responsible for fatty acid biosynthesis in the narazuke strain MS-5. [file aem.01730-25-s0001.pdf]

Supplementary Figure 1

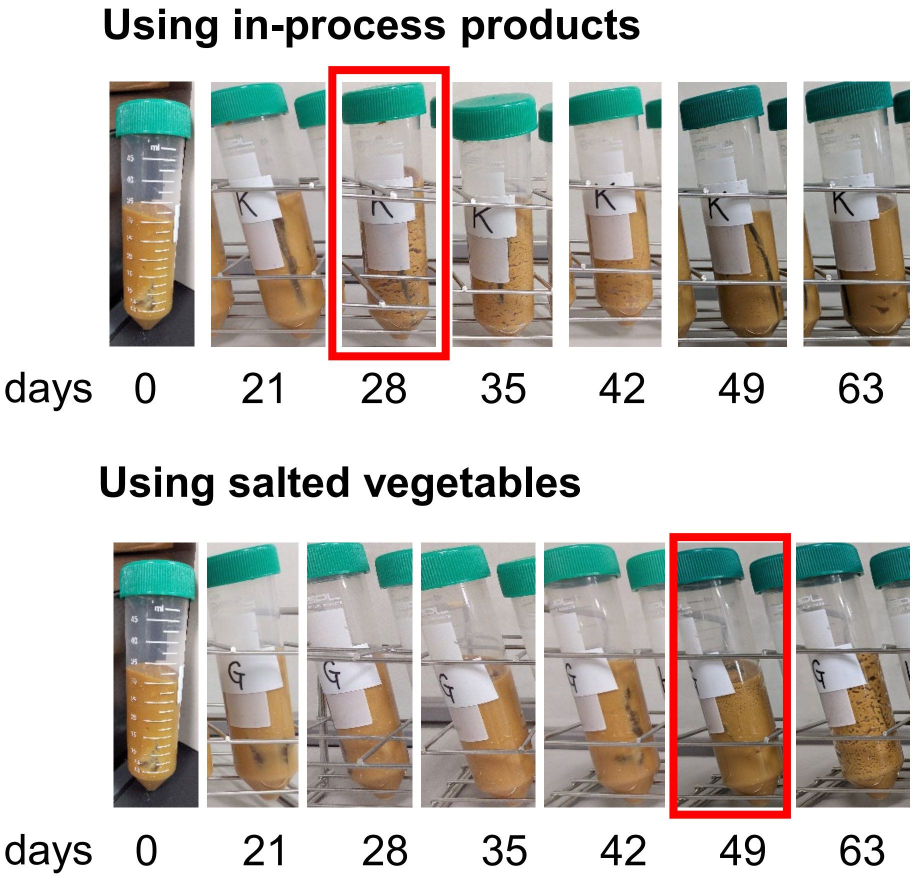

Supplementary Figure 1 Gas generation in the laboratory-scale narazuke fermentation test. Gas was generated at day 28 or at day 49 in the tests when in-process products or salted vegetables was fermented in aged sake kasu, which were highlighted with red frame.

Supplementary Figure 2

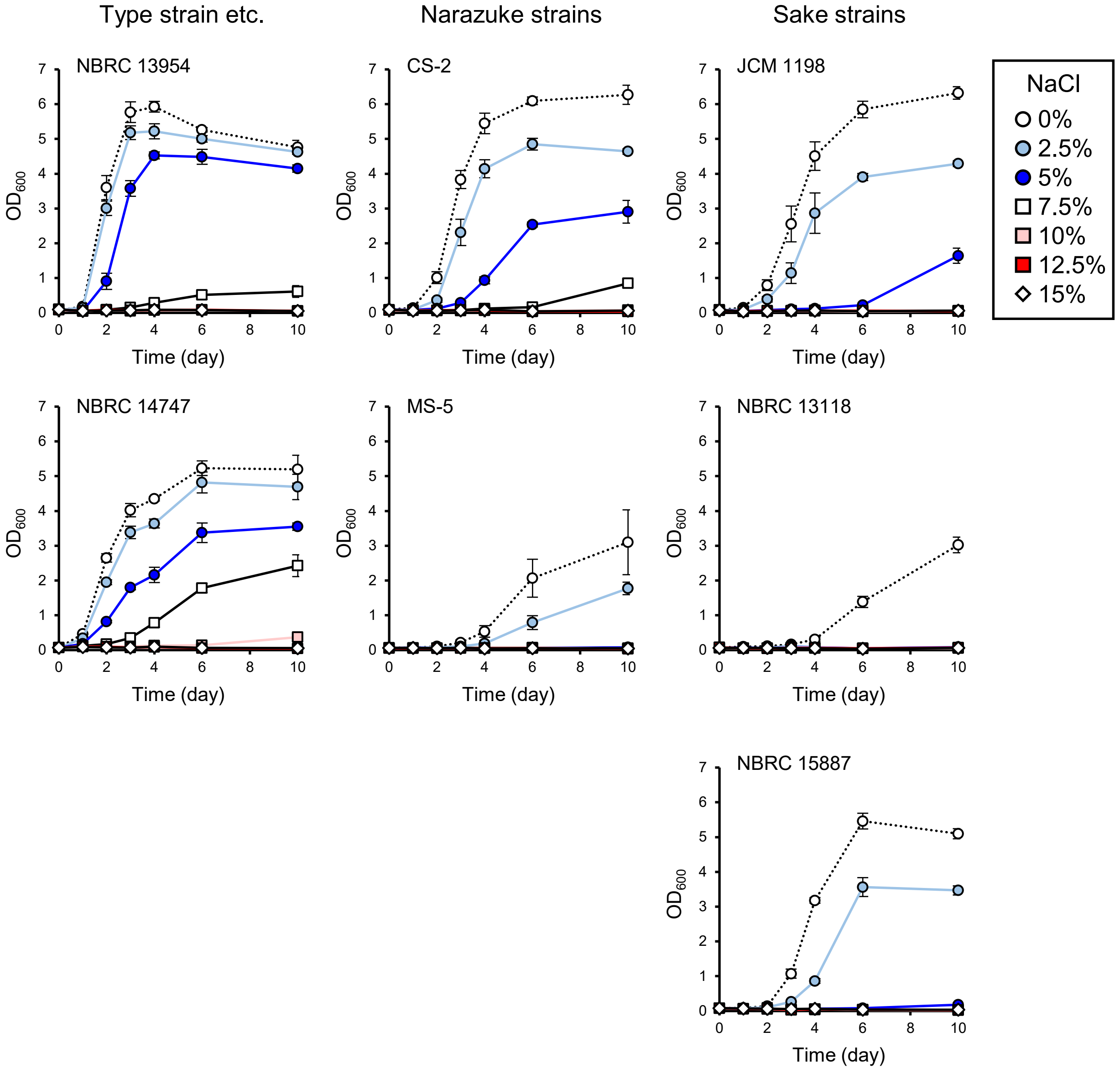

Supplementary Figure 2 Effect of NaCl on growth of various *F. fructivorans* strains. All the strains were anaerobically cultivated in SI media containing various concentrations of NaCl and their growth (OD_{600}) was periodically monitored throughout cultivation. Panels: left, the type strain NBRC 13954^{T} and NBRC 14747 isolated from spoiled salad dressing; middle, narazuke strains (CS-2 and MS-5); and right, sake strains (JCM 1198, NBRC 13118, and NBRC 15887). Symbols: white circles, 0%; light blue circles, 2.5%; blue circles, 5%; white squares, 7.5%; pink squares, 10%; red squares, 12.5%; and white diamonds, 15%. Experiments were conducted in triplicate and error bars indicate standard deviations.

Supplementary Figure 3

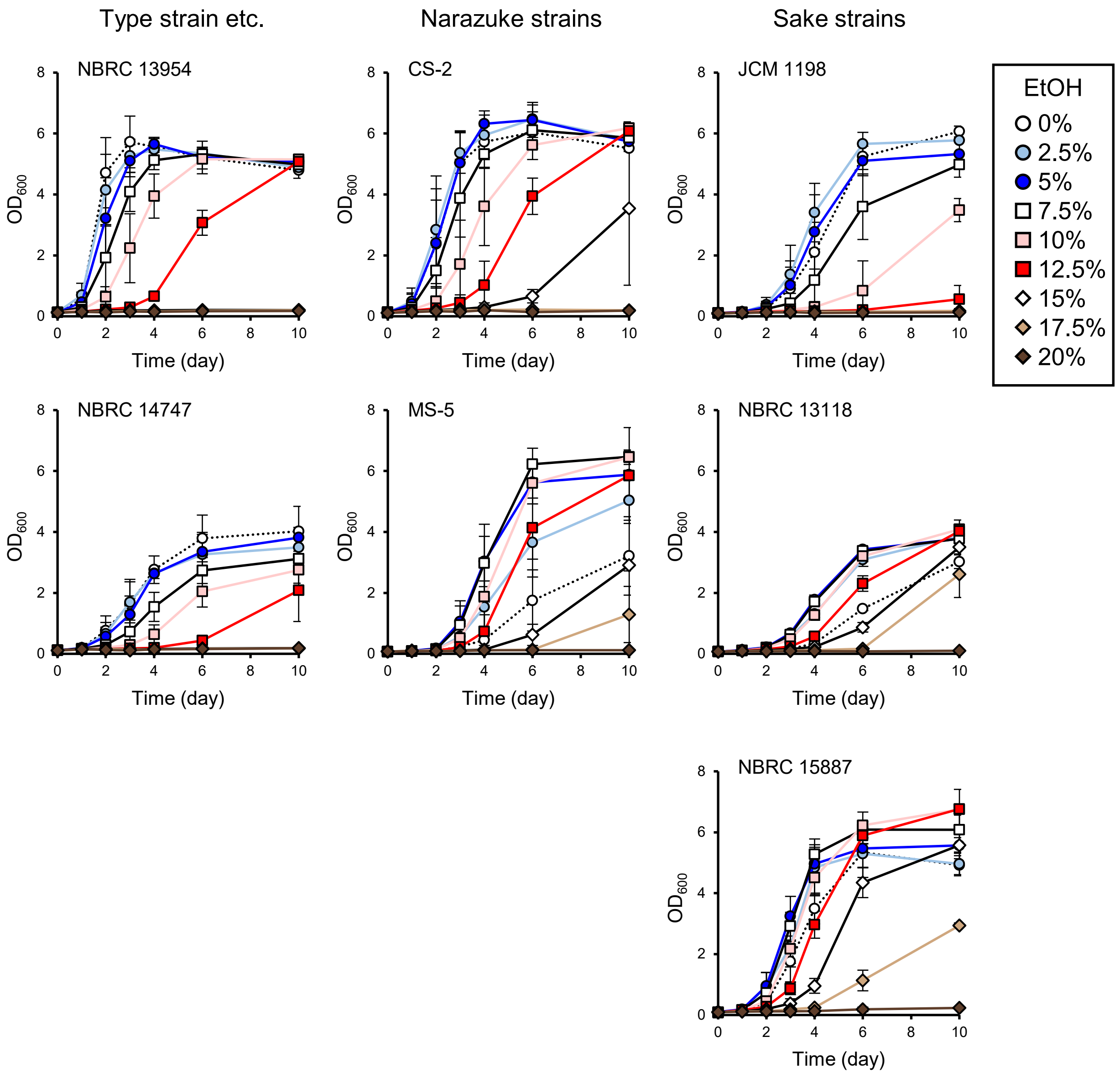

Supplementary Figure 3 Effect of ethanol (EtOH) on growth of various *F. fructivorans* strains. All the strains were anaerobically cultivated in SI media containing various concentrations of EtOH and their growth (OD_{600}) was periodically monitored throughout cultivation. Panels: left, the type strain NBRC 13954^{T} and NBRC 14747 isolated from spoiled salad dressing; middle, narazuke strains (CS-2 and MS-5); and right, sake strains (JCM 1198, NBRC 13118, and NBRC 15887). Symbols: white circles, 0%; light blue circles, 2.5%; blue circles, 5%; white squares, 7.5%; pink squares, 10%; red squares, 12.5%; white diamonds, 15%; light brown diamonds, 17.5%; and brown diamonds, 20%. Experiments were conducted in triplicate and error bars indicate standard deviations. The strains used are the same as those in Supplementary Figure 2.

Supplementary Figure 4

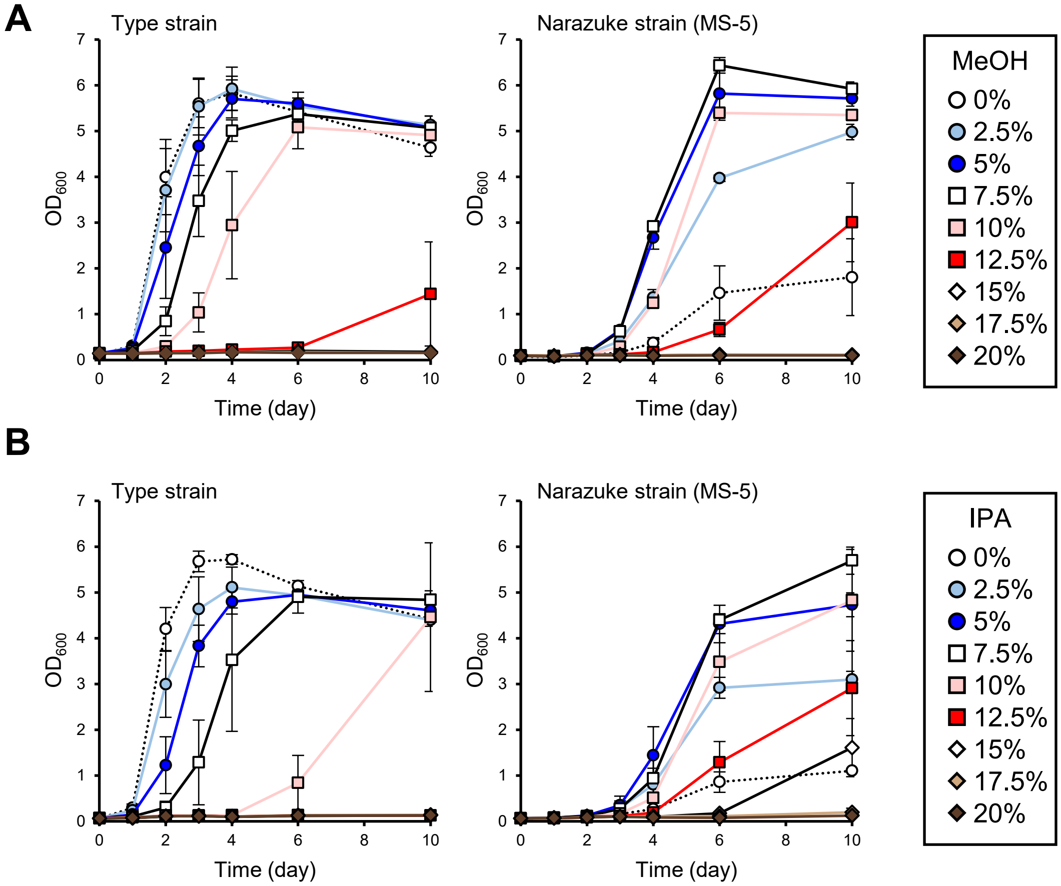

Supplementary Figure 4 Effect of methanol (MeOH) and isopropanol (IPA) on growth of *F. fructivorans* strains. The type strain NBRC 13954^T and the narazuke strain MS-5 were anaerobically cultivated in SI media containing various concentrations of MeOH or IPA and their growth (OD_{600}) were periodically monitored throughout cultivation. (A) Effect of MeOH on growth of the two strains. (B) Effect of IPA on growth of the two strains. (A and B) Panels: left, NBRC 13954^T; and right, MS-5. Symbols: white circles, 0%; light blue circles, 2.5%; blue circles, 5%; white squares, 7.5%; pink squares, 10%; red squares, 12.5%; white diamonds, 15%; light brown diamonds, 17.5%; and brown diamonds, 20%. Experiments were conducted in triplicate and error bars indicate standard deviations.

Supplementary Figure 5

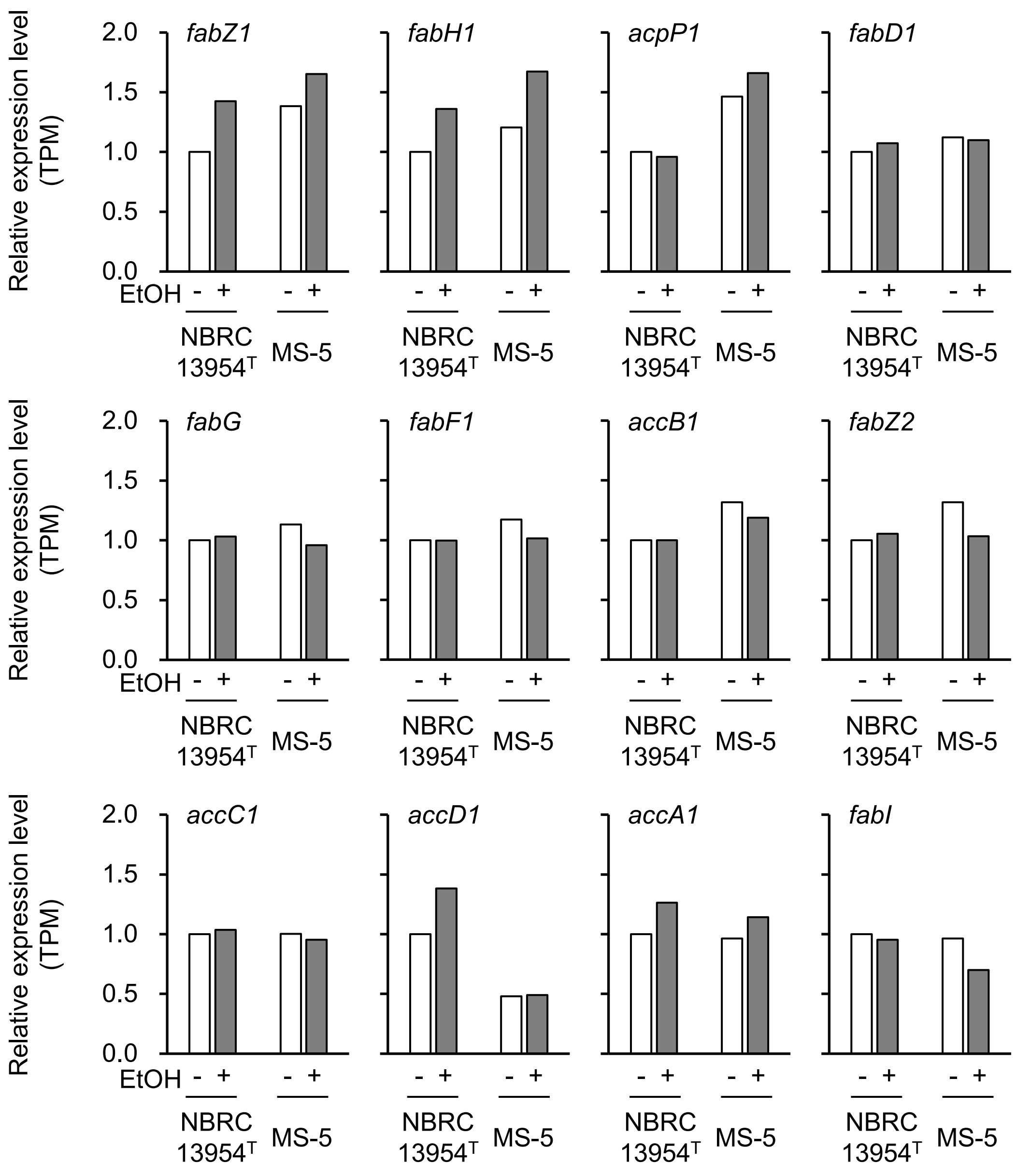

Supplementary Figure 5 Relative expression levels of genes constituting the upstream cluster responsible for fatty acid biosynthesis in the narazuke strain MS-5. The type strain NBRC 13954^T and the narazuke strain MS-5 were cultivated in SI medium supplemented with or without ethanol until the cultures reached the late logarithmic growth phase. Total RNA extracted from the cells were then subjected to mRNA-sequencing as described in the Materials and methods. The expression level of each gene was normalized to that of the type strain grown in the absence of ethanol, which was set to 1. Bars: white, without ethanol; and gray, with ethanol. *fabZ1*, 3-hydroxyacyl-acyl carrier protein (ACP) dehydratase; *fabH1*, β-ketoacyl-ACP synthase III; *acpP1*, ACP; *fabD1*, ACP-*S*-malonyltransferase; *fabG*, 3-oxoacyl-ACP reductase; *fabF1*, β-ketoacyl-ACP synthase II; *accB1*, acetyl-CoA carboxylase biotin carboxyl carrier protein; *fabZ2*, 3-hydroxyacyl-ACP dehydratase; *accC1*, acetyl-CoA carboxylase biotin carboxylase subunit; *accD1*, acetyl-CoA carboxylase carboxyltransferase subunit β; *accA1*, carboxyltransferase subunit α; and *fabI*, enoyl-ACP reductase.
