# Supplementary material for: Ethanolphilic lactic acid bacterium Fructilactobacillus fructivorans as the key microorganism for fermentation of narazuke, a traditional Japanese preserved food
Source: Appl Environ Microbiol. 2025 Nov 18;91(12):e01730-25. doi: 10.1128/aem.01730-25 (PMC12724224; doi:10.1128/aem.01730-25)
Supplement: Tables S1 to S5 — Table S1: The preliminary identification of yeasts isolated from the narazuke samples based on ITS1 sequencing. Table S2: The preliminary identification of bacteria isolated from the narazuke samples based on 16S rRNA gene sequencing. Table S3: The preliminary identification of bacteria isolated in the laboratory-scale narazuke fermentation test (at day 63 after inoculation) based on 16S rRNA gene sequencing. Table S4: The compounds specifically detected in the samples after 63 days of the laboratory-scale narazuke fermentation test. Table S5: Primers used in this study. [file aem.01730-25-s0002.pdf]

Supplementary Table 1 The preliminary identification of yeasts isolated from the narazuke samples based on ITS1 sequencing.

No.	Factory	Vegetable	Sample	Media	Species
1	N	-	Fresh sake kasu	YPD	*Saccharomyces cerevisiae*
2	N	-	Fresh sake kasu	YPD	*Saccharomyces cerevisiae*
3	N	-	Fresh sake kasu	YPD	*Saccharomyces cerevisiae*
4	N	-	Fresh sake kasu	YPD	*Saccharomyces cerevisiae*
5	N	-	Fresh sake kasu	YPD	*Saccharomyces cerevisiae*
6	N	-	Aged sake kasu	YPD	*Starmerella* sp.
7	N	-	Aged sake kasu	YPD	*Starmerella* sp.
8	N	-	Aged sake kasu	YPD	*Starmerella* sp.
9	N	-	Aged sake kasu	YPD	*Starmerella* sp.
10	N	-	Aged sake kasu	YPD	*Starmerella* sp.
11	N	Cucumber	Salted vegetable	YPD	*Zygosaccharomyces rouxii*
12	N	Cucumber	Salted vegetable	YPD	*Zygosaccharomyces rouxii*
13	N	Cucumber	Salted vegetable	YPD	*Zygosaccharomyces rouxii*
14	N	Cucumber	Salted vegetable	YPD	*Zygosaccharomyces rouxii*
15	N	Cucumber	Salted vegetable	YPD	*Wickerhamomyces subpelliculosus*
16	M	Gourd	Salted vegetable	YPD	*Zygosaccharomyces rouxii*
17	M	Gourd	Salted vegetable	YPD	*Zygosaccharomyces rouxii*
18	M	Gourd	Salted vegetable	YPD	*Wickerhamiella versatilis*
19	M	Gourd	Salted vegetable	YPD	*Zygosaccharomyces rouxii*
20	N	Gourd	In process product (Early stage)	YPD	*Starmerella* sp.
21	N	Gourd	In process product (Early stage)	YPD	*Zygosaccharomyces rouxii*
22	N	Gourd	In process product (Early stage)	YPD	*Zygosaccharomyces rouxii*

23	N	Gourd	In process product (Early stage)	YPD	*Zygosaccharomyces rouxii*
24	N	Gourd	In process product (Early stage)	YPD	*Starmerella* sp.
25	N	Gourd	In process product (Late stage)	YPD	*Zygosaccharomyces rouxii*
26	N	Gourd	In process product (Late stage)	YPD	*Zygosaccharomyces rouxii*
27	N	Gourd	In process product (Late stage)	YPD	*Zygosaccharomyces rouxii*
28	N	Gourd	In process product (Late stage)	YPD	*Zygosaccharomyces rouxii*
29	N	Gourd	In process product (Late stage)	YPD	*Zygosaccharomyces rouxii*

Supplementary Table 2 The preliminary identification of bacteria isolated from the narazuke samples based on 16S rRNA gene sequencing.

No.	Factory	Vegetable	Sample	Media	Species
1	M	Gourd	Salted vegetable	MRS	*Streptococcus* sp.
2	M	Gourd	Salted vegetable	MRS	*Streptococcus* sp.
3	M	Gourd	Salted vegetable	MRS	*Streptococcus* sp.
4	M	Gourd	Salted vegetable	MRS	*Streptococcus* sp.
5	N	Gourd	In process product (Late stage)	MRS	*Fructilactobacillus fructivorans*
6	N	Gourd	In process product (Late stage)	MRS	*Fructilactobacillus fructivorans*
7	N	Gourd	In process product (Late stage)	MRS	*Bacillus* sp.
8	N	Gourd	In process product (Late stage)	MRS	*Fructilactobacillus fructivorans*
9	N	Gourd	In process product (Late stage)	MRS	*Bacillus* sp.
10	M	Gourd	In process product (Late stage)	MRS	*Lacticaseibacillus* sp.
11	M	Gourd	In process product (Late stage)	MRS	*Lacticaseibacillus* sp.
12	M	Gourd	In process product (Late stage)	MRS	*Lacticaseibacillus* sp.

13	M	Gourd	In process product (Late stage)	MRS	*Lacticaseibacillus* sp.
14	N	Gourd	Final product	MRS	*Bacillus* sp.
15	N	Gourd	Final product	MRS	*Bacillus* sp.
16	N	Gourd	Final product	MRS	*Bacillus* sp.
17	N	Gourd	Final product	MRS	*Bacillus* sp.
18	N	Gourd	Final product	MRS	*Fructilactobacillus fructivorans*
19	M	Celery	Final product	MRS	*Lacticaseibacillus* sp.
20	M	Celery	Final product	MRS	*Lacticaseibacillus* sp.
21	M	Celery	Final product	MRS	*Lacticaseibacillus* sp.
22	M	Celery	Final product	MRS	*Lacticaseibacillus* sp.
23	M	Gourd	In process product (Early stage)	SI	*Fructilactobacillus fructivorans*
24	M	Gourd	In process product (Early stage)	SI	*Fructilactobacillus fructivorans*
25	M	Gourd	In process product (Early stage)	SI	*Fructilactobacillus fructivorans*
26	M	Gourd	In process product (Early stage)	SI	*Fructilactobacillus fructivorans*
27	N	Gourd	In process product (Late stage)	SI	*Fructilactobacillus fructivorans*
28	N	Gourd	In process product (Late stage)	SI	*Fructilactobacillus fructivorans*

29	N	Gourd	In process product (Late stage)	SI	*Fructilactobacillus fructivorans*
30	N	Gourd	In process product (Late stage)	SI	*Fructilactobacillus fructivorans*
31	N	Gourd	In process product (Late stage)	SI	*Fructilactobacillus fructivorans*
32	M	Gourd	In process product (Late stage)	SI	*Fructilactobacillus fructivorans*
33	M	Gourd	In process product (Late stage)	SI	*Fructilactobacillus fructivorans*
34	M	Gourd	In process product (Late stage)	SI	*Fructilactobacillus fructivorans*
35	M	Gourd	In process product (Late stage)	SI	*Fructilactobacillus fructivorans*
36	N	Gourd	Final product	SI	*Fructilactobacillus fructivorans*
37	N	Gourd	Final product	SI	*Fructilactobacillus fructivorans*
38	N	Gourd	Final product	SI	*Fructilactobacillus fructivorans*
39	N	Gourd	Final product	SI	*Fructilactobacillus fructivorans*
40	N	Gourd	Final product	SI	*Fructilactobacillus fructivorans*
41	M	Gourd	Final product	SI	*Fructilactobacillus fructivorans*

42	M	Gourd	Final product	SI	*Fructilactobacillus fructivorans*
43	M	Gourd	Final product	SI	*Fructilactobacillus fructivorans*
44	M	Gourd	Final product	SI	*Fructilactobacillus fructivorans*
45	M	Carrot	Final product	SI	*Fructilactobacillus fructivorans*
46	M	Carrot	Final product	SI	*Fructilactobacillus fructivorans*
47	M	Carrot	Final product	SI	*Fructilactobacillus fructivorans*
48	M	Carrot	Final product	SI	*Fructilactobacillus fructivorans*
49	M	Celery	Final product	SI	*Fructilactobacillus fructivorans*
50	M	Celery	Final product	SI	*Fructilactobacillus fructivorans*
51	M	Celery	Final product	SI	*Fructilactobacillus fructivorans*
52	M	Celery	Final product	SI	*Fructilactobacillus fructivorans*

Supplementary Table 3 The preliminary identification of bacteria isolated in the laboratory-scale narazuke fermentation test (at day 63 after inoculation) based on 16S rRNA gene sequencing.

No.	Species
1	*Fructilactobacillus fructivorans*
2	*Fructilactobacillus fructivorans*
3	*Fructilactobacillus fructivorans*
4	*Fructilactobacillus fructivorans*
5	*Fructilactobacillus fructivorans*
6	*Fructilactobacillus fructivorans*
7	*Fructilactobacillus fructivorans*
8	*Fructilactobacillus fructivorans*
9	*Fructilactobacillus fructivorans*
10	*Fructilactobacillus fructivorans*
11	*Fructilactobacillus fructivorans*
12	*Fructilactobacillus fructivorans*
13	*Fructilactobacillus fructivorans*
14	*Fructilactobacillus fructivorans*
15	*Fructilactobacillus fructivorans*

Supplementary Table 4 The compounds specifically detected in the samples after 63 days of the laboratory-scale narazuke fermentation test.

Compound	PubChem CID	Day 0	Day 63
3-Hydroxypicolinic acid	13401	N.D.	8.2E-05
3-Methoxy-4-hydroxyphenylethyleneglycol	10805	N.D.	3.8E-04
4-Hydroxyhippuric acid	151012	N.D.	1.4E-04
7-Amino-4-hydroxy-2-naphthalenesulfonic acid	6868	N.D.	7.4E-05
8-Hydroxyoctanoic acid-1	69820	N.D.	4.7E-04
Adenosine	60961	N.D.	1.2E-02
Azetidine 2-carboxylic acid	16486	N.D.	9.1E-04
Crotonic acid	637090	N.D.	1.8E-03
Diethanolamine	8113	N.D.	1.9E-03
Glutathione (GSH)	124886	N.D.	1.1E-04
Glutathione	11161	N.D.	1.0E-03
Guanosine	6802	N.D.	3.8E-04
Imidazole-4-methanol	1745	N.D.	8.5E-05
Inosine	6021	N.D.	2.5E-04
Maleic acid	444266	N.D.	1.8E-04
Met-Val-Pro	10291254	N.D.	1.1E-03
N-Methylalanine	5288725	N.D.	5.4E-04
N^6-($\Delta 2$-Isopentenyl)adenine	92180	N.D.	3.3E-04
Propionic acid	1032	N.D.	2.2E-03
S-Adenosylmethionine	34755	N.D.	2.8E-04
S-Carboxymethylcysteine	193653	N.D.	4.7E-05

Sebacic acid	5192	N.D.	1.4E-04
Spectinomycin_+H2O	15541	N.D.	6.0E-05
Spermine	1103	N.D.	4.6E-05
γ-Glu-Cys	123938	N.D.	1.2E-04
Lactic acid	612	3.4E-01	4.0E+00

Supplementary Table 5 Primers used in this study.

Primer	Sequence (5' – 3')	Use
ITS1F-KYO1	CTHGGTCATTTAGAGGAASTAA	Identification of fungi
ITS2-KYO2	TTYRCTRCGTTCTTCATC	Identification of fungi
ITS_1F	GTAACAAGGTYTCCGT	Identification of fungi
ITS_R1	CGTTCTTCATCGATG	Identification of fungi
515f	GTGCCAGCMGCCGCGGTAA	Identification of bacteria/archaea
806r	GGACTACHVGGGTWTCTAAT	Identification of bacteria/archaea
PC3mod	GGACTAHAGGGTATCTAAT	Identification of bacteria/archaea
27f	AGAGTTTGATCMTGGCTCAG	Identification of bacteria/archaea
1492f	TACGGYTACCTTGTTACGACTT	Identification of bacteria/archaea
accC1F	CGTTGTAACTAGGGATGGAC	Genotyping of *accC1*
accC1R	CTGGATAGTTCATCAGAACTC	Genotyping of *accC1*
accC2F	AAGAGCGGCTCAATGGTTGAG	Genotyping of *accC2*
accC2R	TGAACCCAAATGTGATCAGG	Genotyping of *accC2*
